# Supplementary figures and images for: Moving north: Warmer waters expand populations of deep-water cartilaginous fishes into Arctic waters
Source: PLoS One. 2026 Mar 5;21(3):e0343778. doi: 10.1371/journal.pone.0343778 (PMC12962514; doi:10.1371/journal.pone.0343778)

**
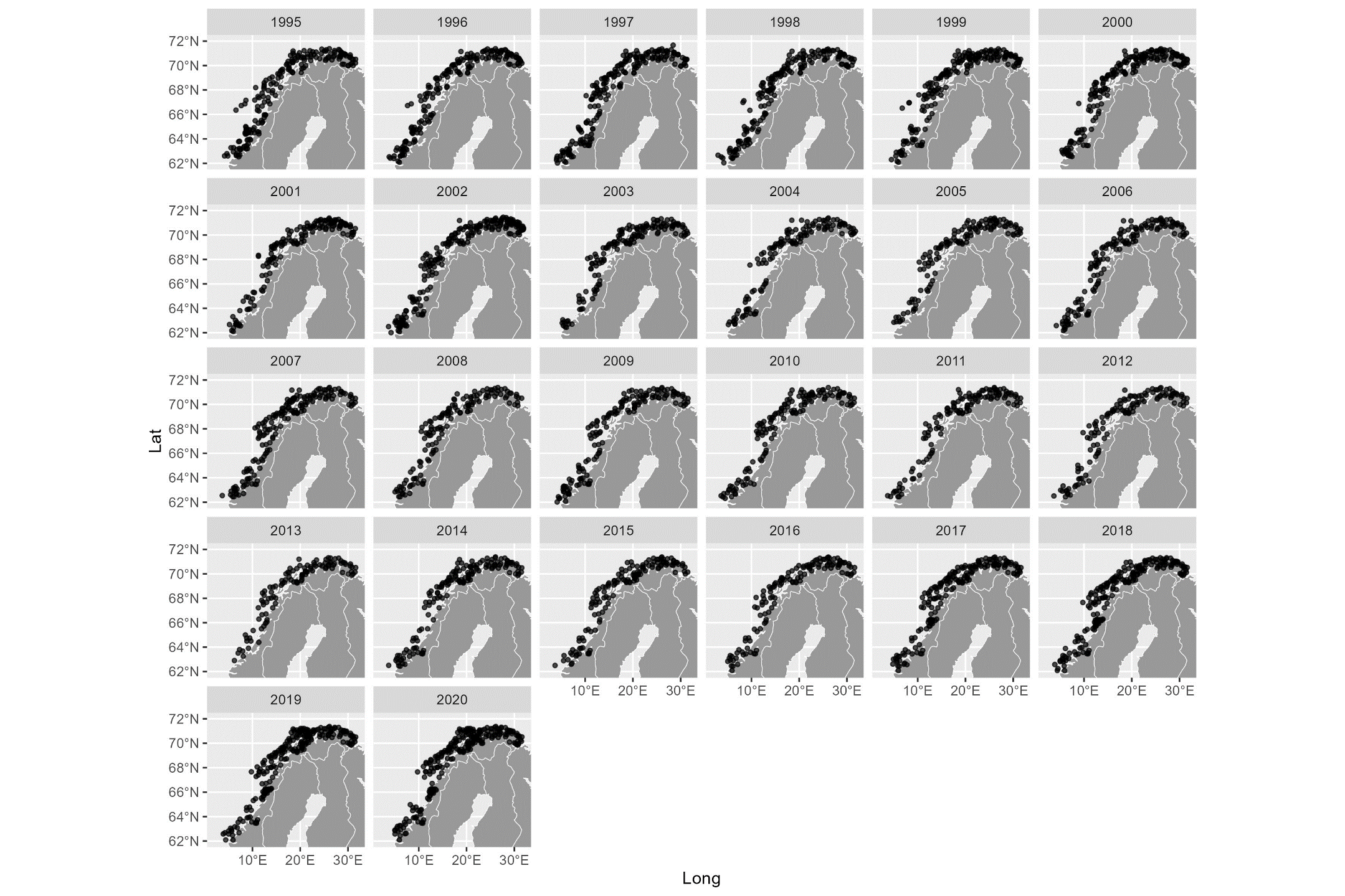
**

Supplement: S1 Fig — (DOCX) [file pone.0343778.s001.docx]

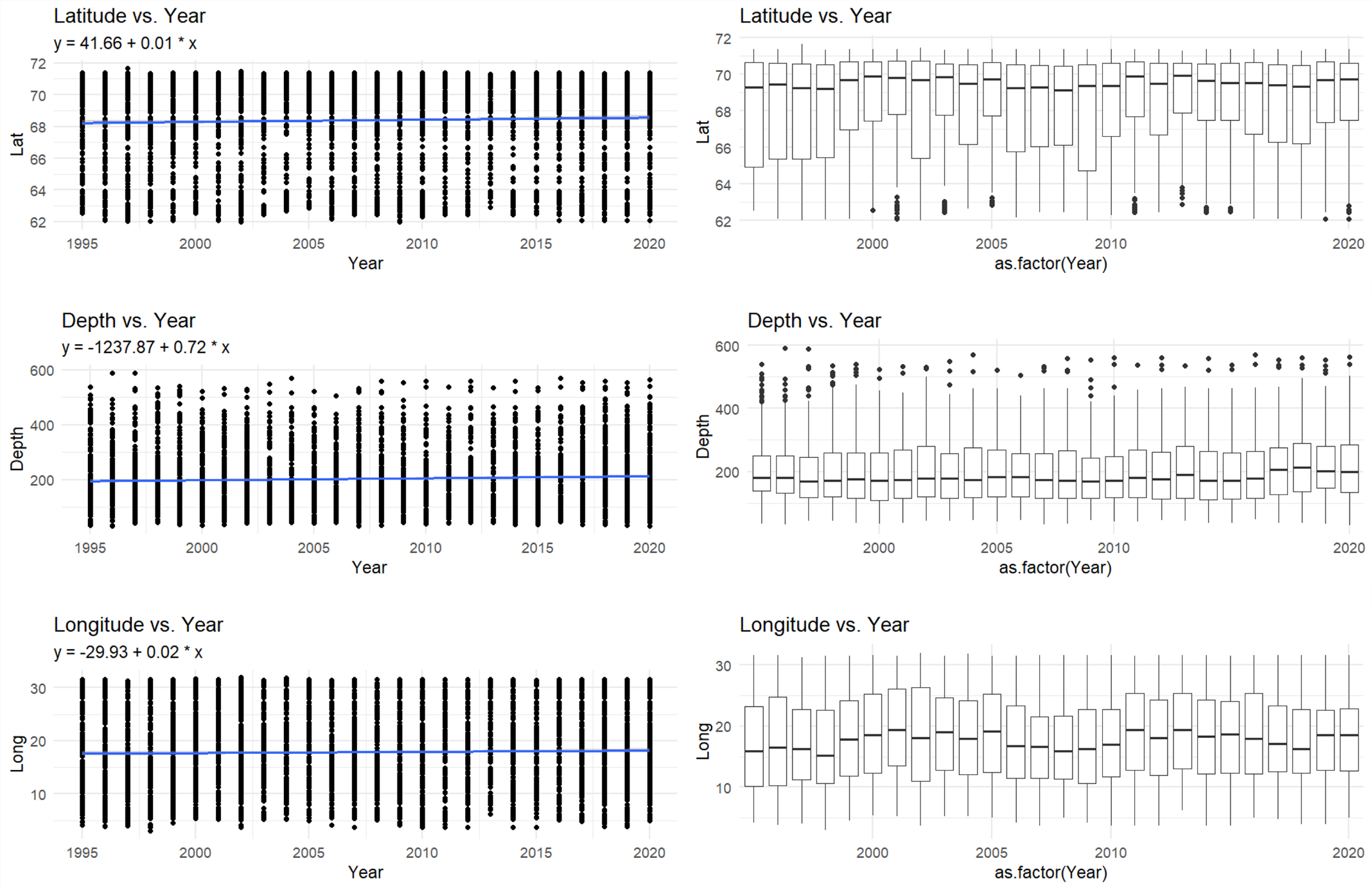

Supplement: S2 Fig — (DOCX) [file pone.0343778.s002.docx]

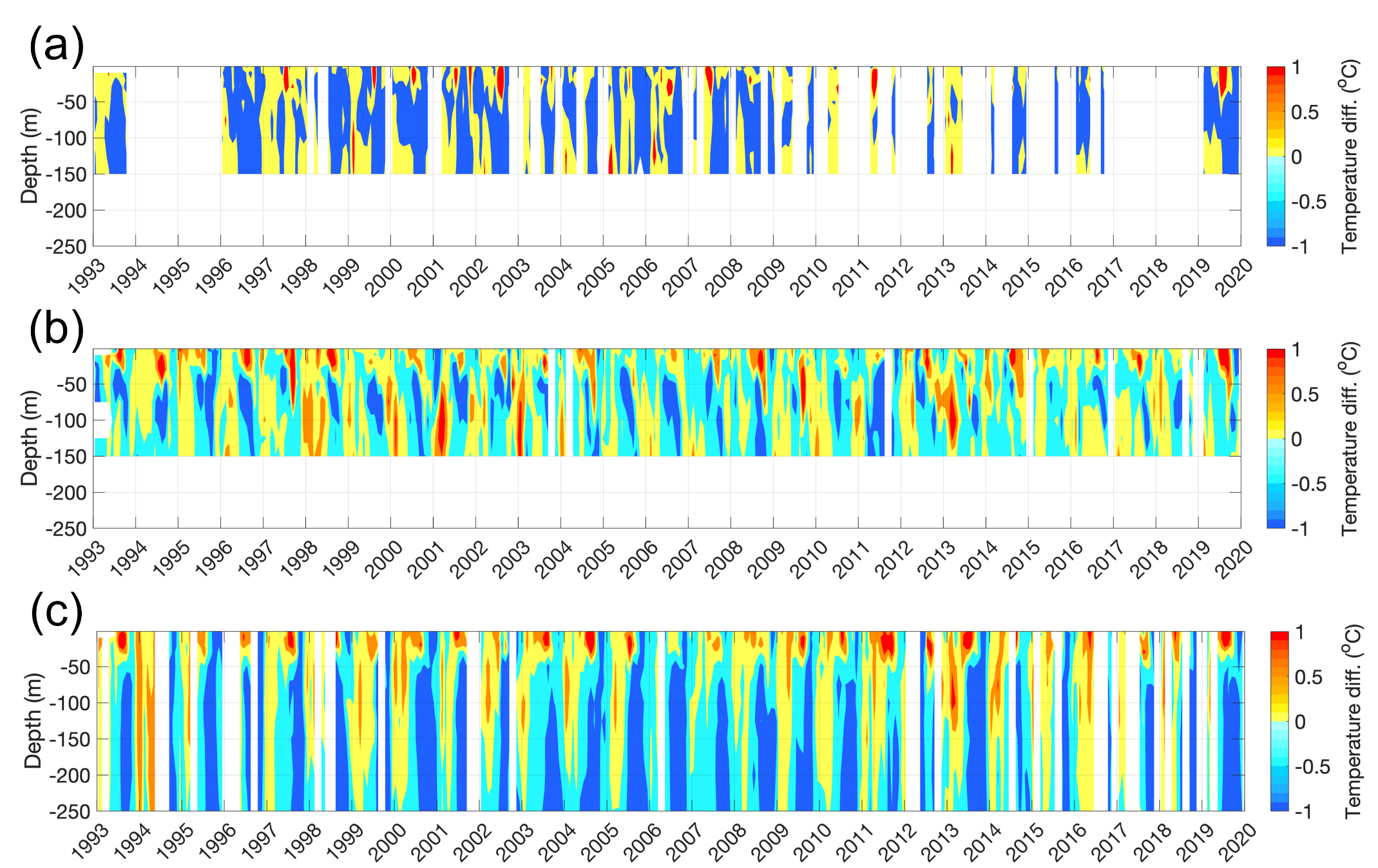

Supplement: S3 Fig — Temperature difference above 0 °C means that the model is warmer than the observations. (DOCX) [file pone.0343778.s003.docx]

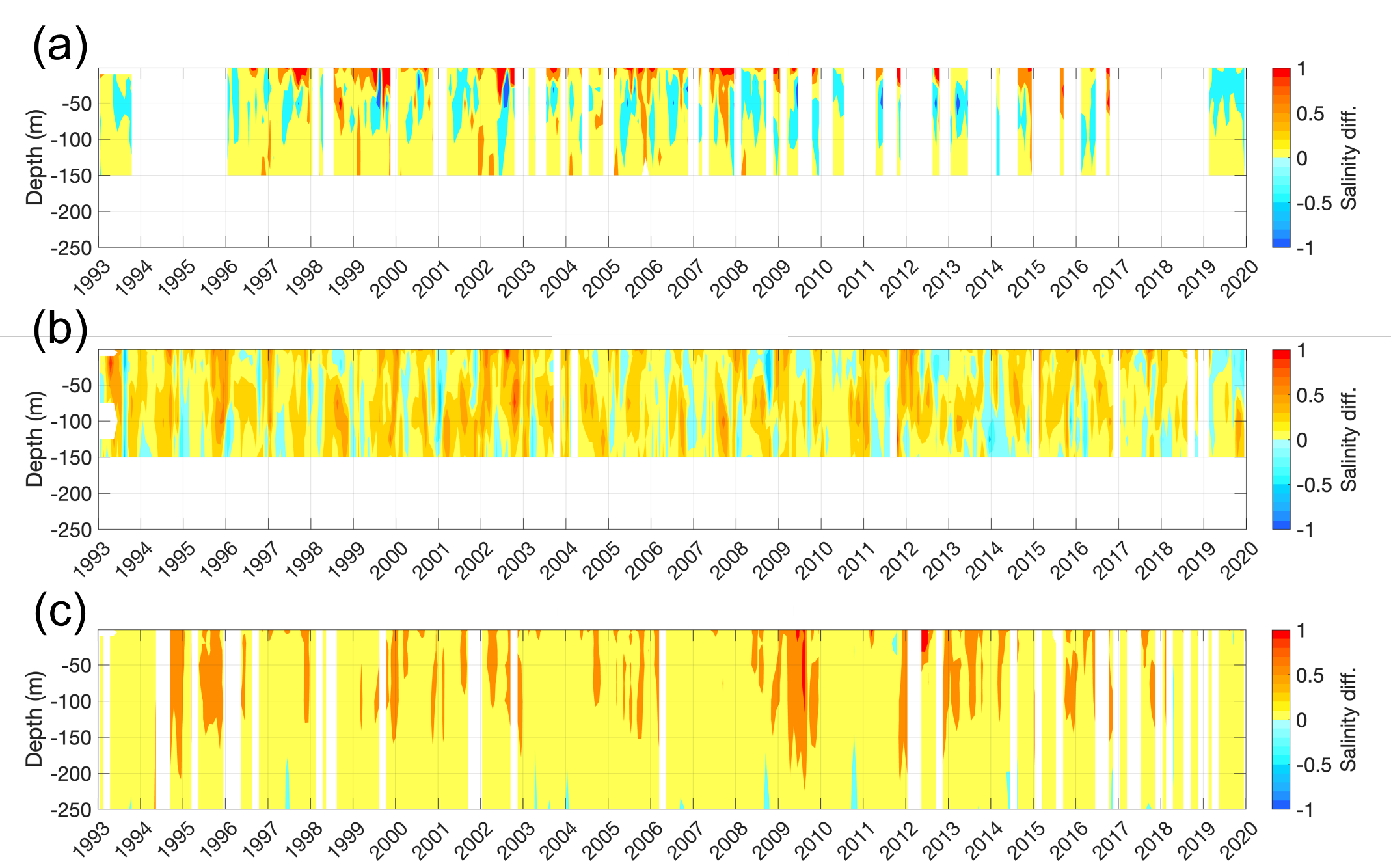

Supplement: S4 Fig — Salinity difference above 0 means that the model is more saline than the observations. (DOCX) [file pone.0343778.s004.docx]

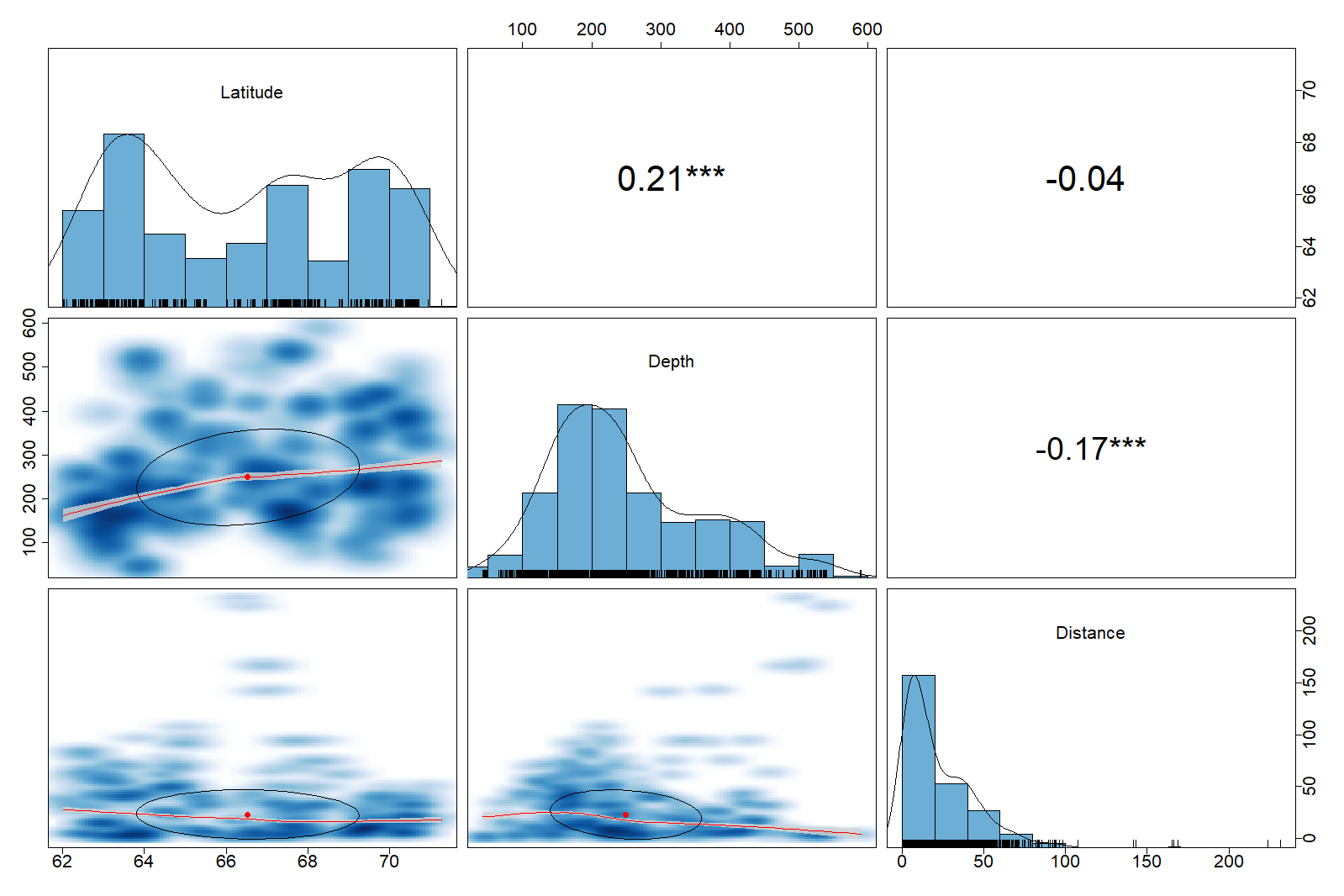

Supplement: S5 Fig — (DOCX) [file pone.0343778.s005.docx]

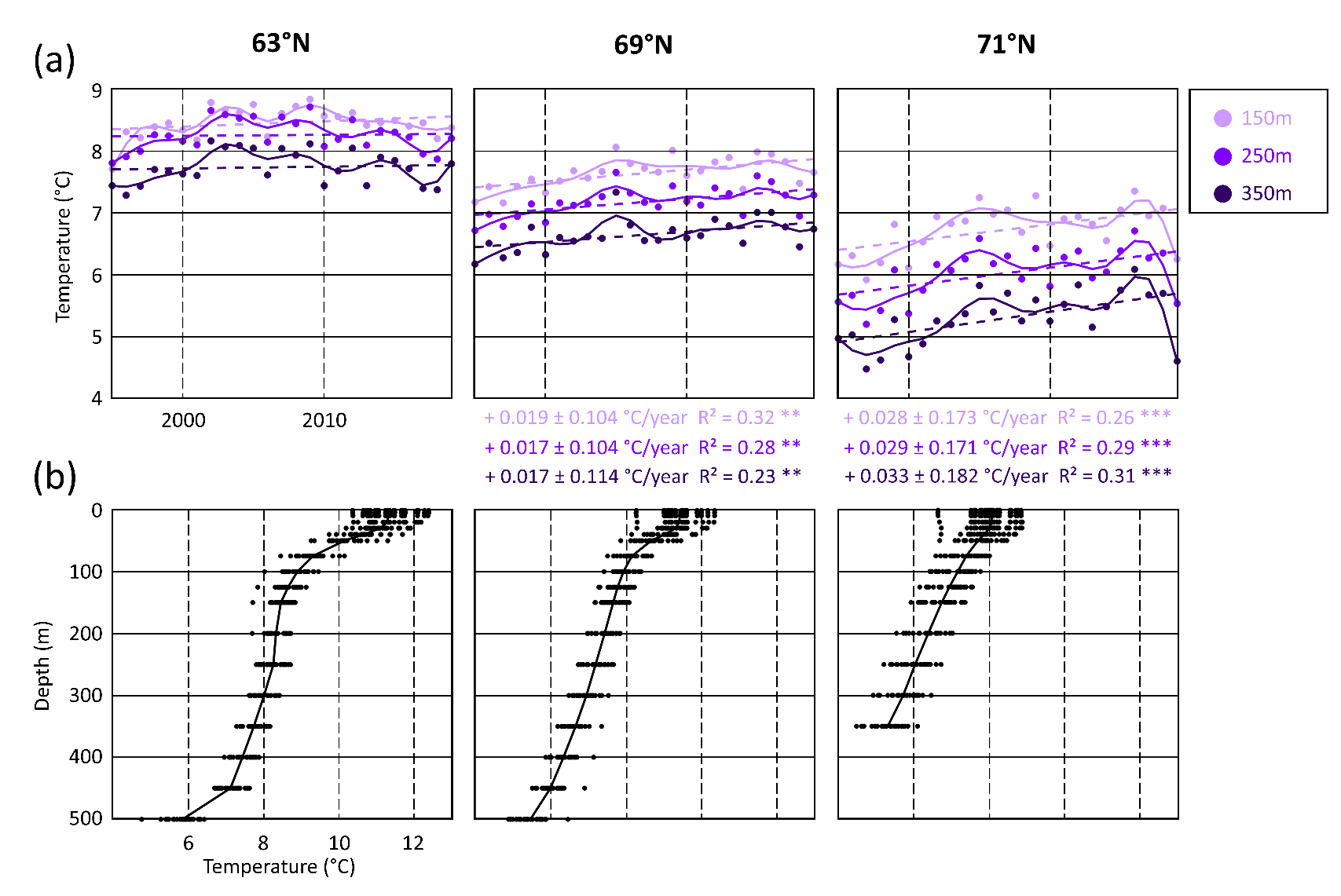

Supplement: S6 Fig — (a) shows each year’s temperatures as dot, with lowpass-filtered time series using 5-year cut-off period as solid lines and the linear trend line as dashed lines. The colours separate the three depths used. Only the linear slopes with a significant p-value are shown. Change through time is written as: slope ± SE. Statistical significance levels are given as ***: p < 0.001, **: p < 0.01, *: p < 0.05. (b) show autumn temperature with depth for all years (dots) where the line denotes the 1995–2020 average. (DOCX) [file pone.0343778.s006.docx]

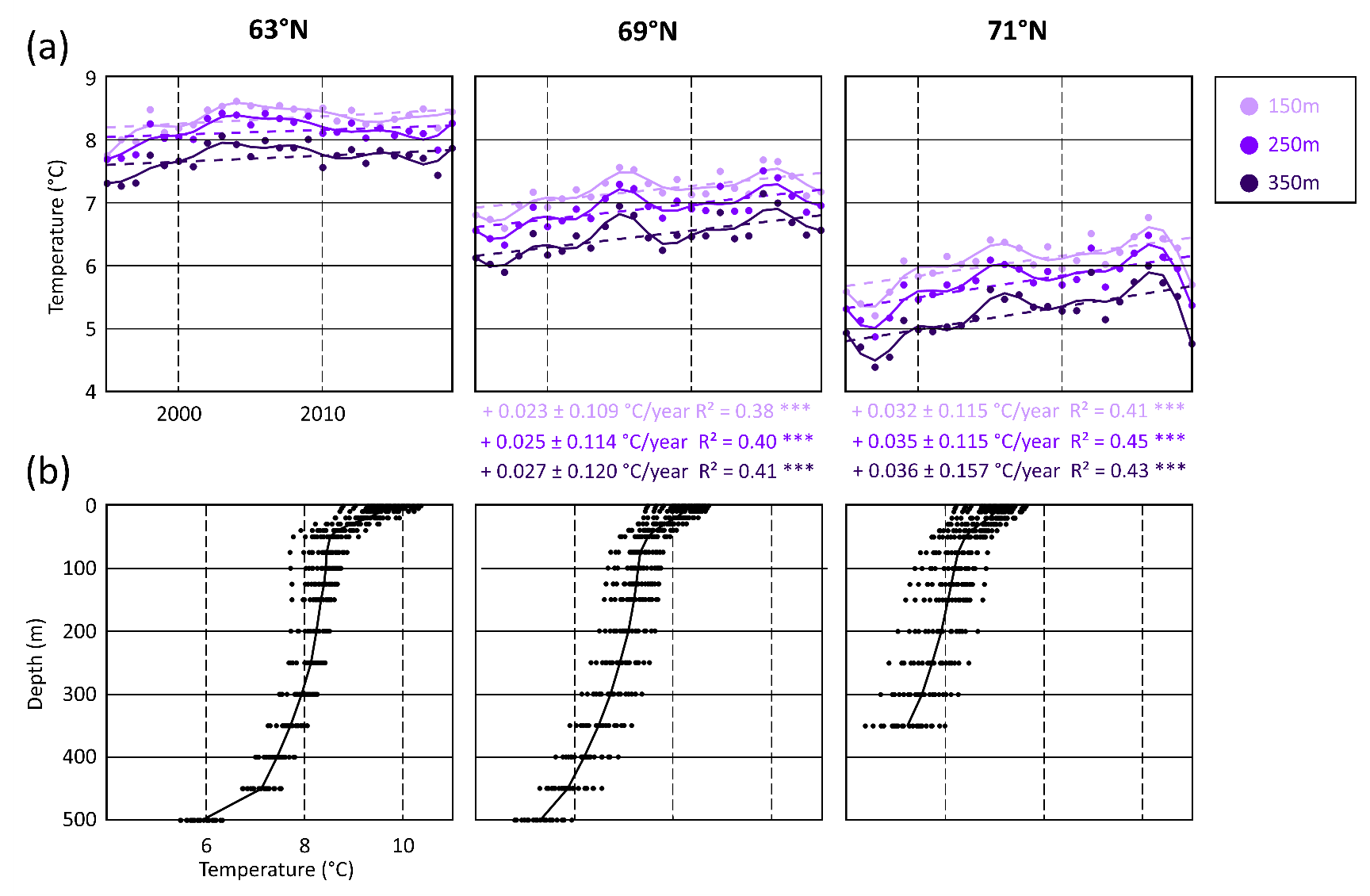

Supplement: S7 Fig — (a) shows each year’s temperatures as dot, lowpass-filtered time series using 5-year cut-off period as solid lines and the linear trend line as dashed lines. The colours separate the three depths used, which is 150, 250 and 350 m. Only the slopes with a significant p-value are shown. Change through time is written as: slope ± SE. Statistical significance levels are given as ***: p < 0.001, **: p < 0.01, *: p < 0.05. (b) shows entire year temperature with depth for all years (dots) where the line denotes the 1995–2020 average. (DOCX) [file pone.0343778.s007.docx]

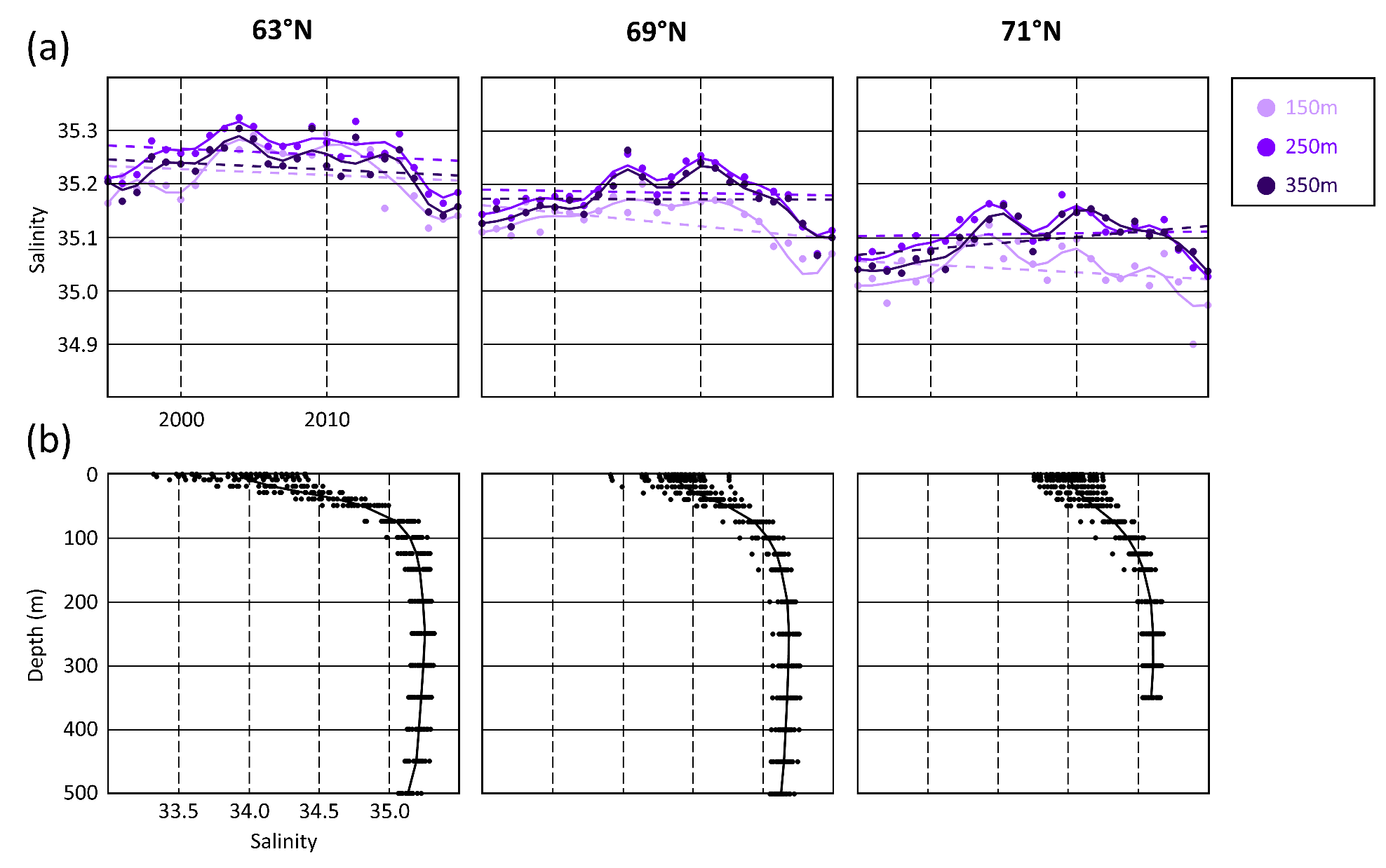

Supplement: S8 Fig — (a) shows each year’s salinities as dot, lowpass-filtered time series using 5-year cut-off period as solid lines and the linear trend line as dashed lines. The colours separate the three depths used, which is 150, 250 and 350 m. No slopes have a significant p-value. (b) shows autumn salinity with depth for all years (dots) where the line denotes the 1995–2020 average. (DOCX) [file pone.0343778.s008.docx]

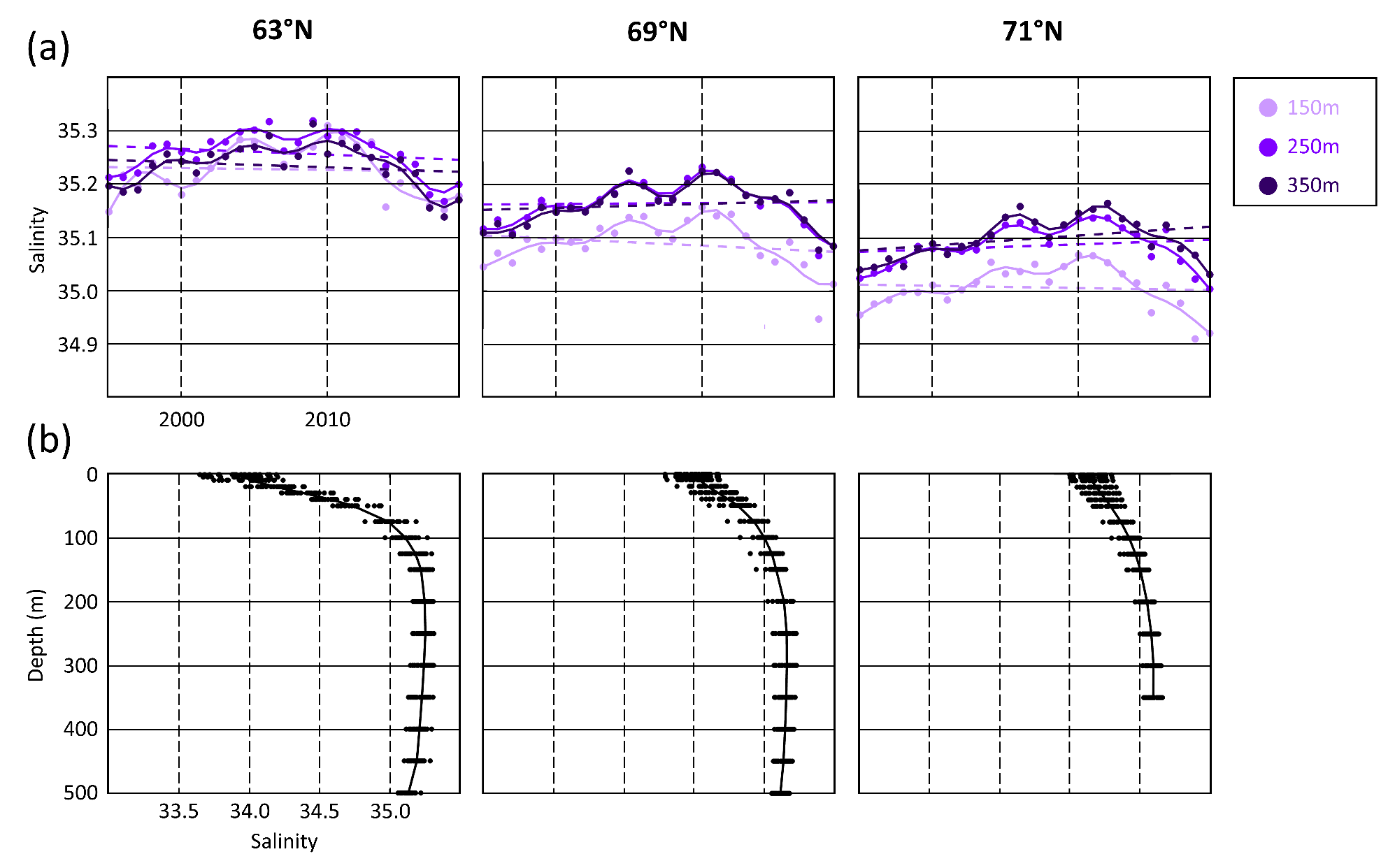

Supplement: S9 Fig — (a) shows each year’s salinities as dot, lowpass-filtered time series using 5-year cut-off period as solid lines and the linear trend line as dashed lines. The colours separate the three depths used, which is 150, 250 and 350 m. No slopes have a significant p-value. (b) shows entire year salinity with depth for all years (dots) where the line denotes the 1995–2020 average. (DOCX) [file pone.0343778.s009.docx]

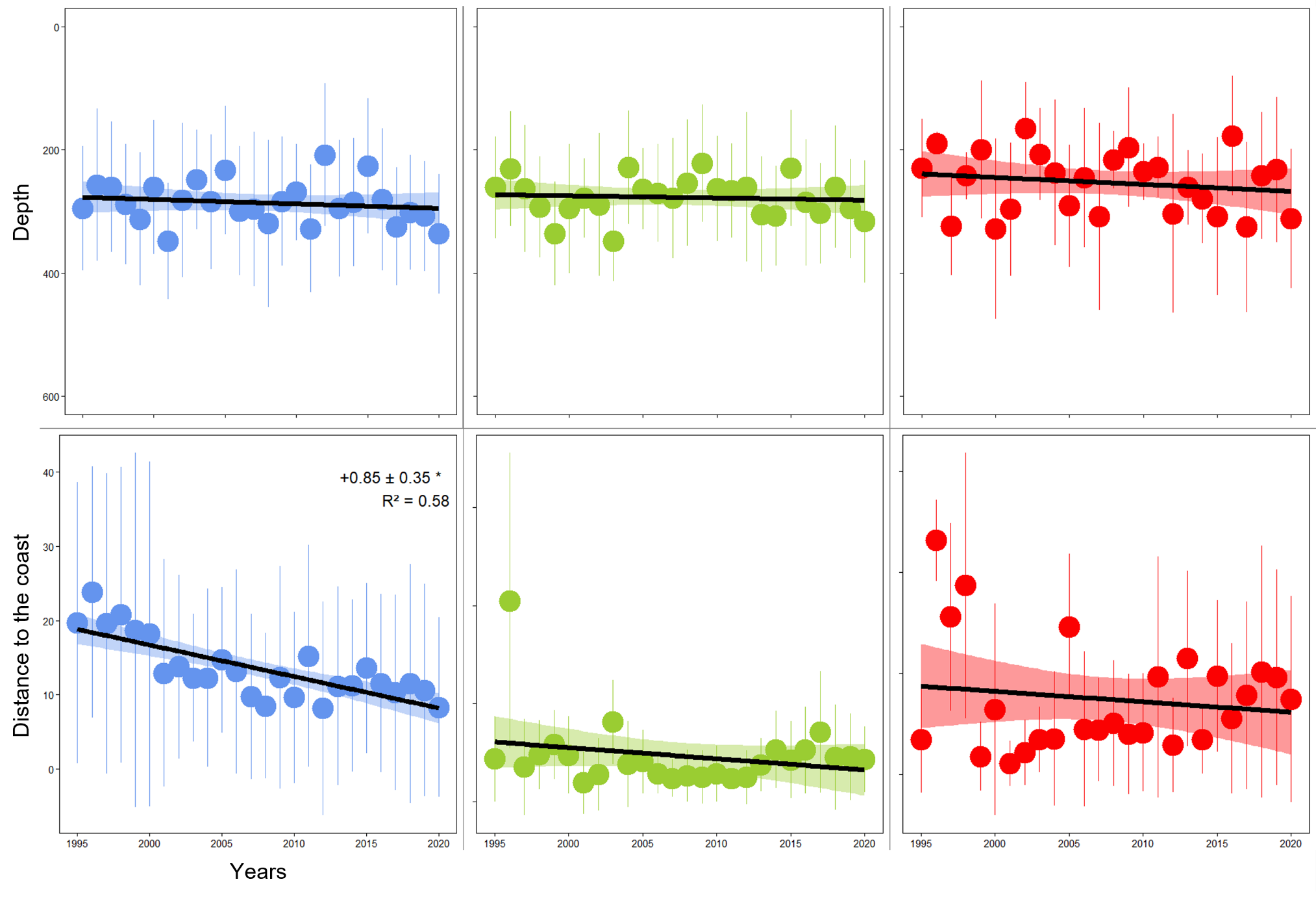

Supplement: S10 Fig — Change through time is written as: slope ± SE. Significant value of statistical tests given as *: p < 0.05. (DOCX) [file pone.0343778.s010.docx]
