# Supplementary material for: Moving north: Warmer waters expand populations of deep-water cartilaginous fishes into Arctic waters
Source: PLoS One. 2026 Mar 5;21(3):e0343778. doi: 10.1371/journal.pone.0343778 (PMC12962514; doi:10.1371/journal.pone.0343778)
Supplement: S1 Table — (DOCX) [file pone.0343778.s011.docx]

| **Years** | **Number of trawl stations** | **Dates** | **Depth range**  **(m)** | **Latitude range**  **(°C)** | **Species occurrence (%)** | | |
| --- | --- | --- | --- | --- | --- | --- | --- |
|  |  |  |  |  | *Chimaera monstrosa* | *Etmopterus spinax* | *Galeus melastomus* |
| 1995 | 151 | 09/13-11/04 | 34-539 | 62.6-71.4 | 31.1 | 12.6 | 13.9 |
| 1996 | 150 | 09/11-10/28 | 32-590 | 62.4-71.4 | 30.0 | 1.3 | 2.7 |
| 1997 | 149 | 08/20-10/26 | 43-588 | 62.0-71.7 | 24.2 | 11.4 | 13.4 |
| 1998 | 147 | 10/14-11/18 | 44-536 | 62.0-71.4 | 28.6 | 15.0 | 14.3 |
| 1999 | 152 | 10/08-11/18 | 37-540 | 62.3-71.4 | 32.9 | 11.2 | 9.2 |
| 2000 | 152 | 10/15-11/16 | 46-523 | 62.7-71.4 | 30.9 | 15.8 | 10.5 |
| 2001 | 109 | 10/15-11/17 | 36-532 | 62.7-71.4 | 25.7 | 16.5 | 9.2 |
| 2002 | 168 | 10/12-11/25 | 43-531 | 62.0-71.5 | 24.4 | 13.7 | 9.5 |
| 2003 | 100 | 10/11-11/14 | 43-520 | 62.5-71.3 | 28.0 | 14.0 | 8.0 |
| 2004 | 91 | 10/13-11/10 | 45-518 | 62.7-71.4 | 37.4 | 15.4 | 17.6 |
| 2005 | 96 | 10/11-11/08 | 44-521 | 62.9-71.4 | 30.2 | 16.7 | 8.3 |
| 2006 | 107 | 10/25-11/19 | 45-505 | 62.5-71.4 | 39.3 | 19.6 | 16.8 |
| 2007 | 129 | 10/16-11/19 | 35-532 | 62.5-71.4 | 35.7 | 17.8 | 12.4 |
| 2008 | 102 | 10/30-12/15 | 34-522 | 62.4-71.4 | 44.1 | 20.6 | 20.6 |
| 2009 | 114 | 09/29-12/05 | 45-491 | 62.0-71.4 | 43.0 | 15.8 | 20.2 |
| 2010 | 110 | 09/28-11/09 | 40-539 | 62.3-71.4 | 35.5 | 16.4 | 14.5 |
| 2011 | 85 | 10/07-11/10 | 42-537 | 62.5-71.4 | 40.0 | 15.3 | 18.8 |
| 2012 | 89 | 10/02-11/11 | 45-538 | 62.5-71.4 | 40.4 | 18.0 | 16.9 |
| 2013 | 78 | 10/03-11/05 | 44-535 | 62.9-71.3 | 42.3 | 20.5 | 19.2 |
| 2014 | 106 | 09/29-10/31 | 36-521 | 62.5-71.7 | 40.6 | 17.9 | 18.9 |
| 2015 | 95 | 10/04-11/04 | 36-536 | 62.5-71.4 | 44.2 | 23.2 | 23.2 |
| 2016 | 96 | 10/02-10/29 | 46-539 | 62.5-71.4 | 46.9 | 21.9 | 18.8 |
| 2017 | 122 | 10/04-11/13 | 37-538 | 62.5-71.4 | 47.5 | 23.0 | 18.0 |
| 2018 | 142 | 10/05-11/12 | 37-538 | 62.1-71.3 | 50.7 | 23.2 | 26.8 |
| 2019 | 157 | 10/05-11/12 | 35-537 | 62.1-71.4 | 43.3 | 19.7 | 16.6 |
| 2020 | 162 | 10/08-11/13 | 30-540 | 62.6-71.4 | 42.0 | 17.9 | 22.2 |
| TOTAL | 3159 |  |  |  |  |  |  |
