# Supplementary material for: Moving north: Warmer waters expand populations of deep-water cartilaginous fishes into Arctic waters
Source: PLoS One. 2026 Mar 5;21(3):e0343778. doi: 10.1371/journal.pone.0343778 (PMC12962514; doi:10.1371/journal.pone.0343778)
Supplement: S2 Table — (DOCX) [file pone.0343778.s012.docx]

|  |  | **Temperature (°C)**  *From 2.8 to 12.7* | **Depth (m)**  *From 30 to 590* | **Latitude (°N)**  *From 62.0 to 71.7* | **Salinity (psu)**  *From 32.1 to 35.6* | **Distance (km)**  *From 0.1 to 232* |
| --- | --- | --- | --- | --- | --- | --- |
| ***Chimaera monstrosa*** | Habitat occupancy | 4.1 - 12.4 | 42 - 590 | 62.0 - 71.3 | 32.7 - 35.6 | 0.1 - 231 |
|  | Habitat preference | 7.0 - 8.7 | 129 - 387 | 63.8 - 69.8 | 34.5 - 35.5 | 6 -32 |
| ***Etmopterus spinax*** | Habitat occupancy | 5.7 - 11.6 | 35 - 571 | 62.0 - 69.8 | 32.6 - 35.6 | 0.1 - 141 |
|  | Habitat preference | 6.9 - 8.6 | 183 - 407 | 63.8 - 68.1 | 34.4 - 35.6 | 0.1 - 23 |
| ***Galeus melastomus*** | Habitat occupancy | 6.2 – 11.2 | 35 - 407 | 63.4 - 68.5 | 34.5 - 35.6 | 0.1 - 46 |
|  | Habitat preference | 7.2 - 9.1 | 111 - 380 | 63.5 - 65.6 | 34.2 - 35.5 | 3 - 32 |
