# Supplementary material for: Moving north: Warmer waters expand populations of deep-water cartilaginous fishes into Arctic waters
Source: PLoS One. 2026 Mar 5;21(3):e0343778. doi: 10.1371/journal.pone.0343778 (PMC12962514; doi:10.1371/journal.pone.0343778)
Supplement: S3 Table — The green number represent the number of positive co-occurrence while the red corresponds to the negative ones. The percentage of co-occurrence was calculated from the genus. The percentages calculated correspond to the frequency of observation of these species when C. monstrosa, E. spinax and G. melastomus were caught at a station. (DOCX) [file pone.0343778.s013.docx]

| **Group** | **Genus** | ***Chimaera monstrosa***  (45/27) | ***Etmopterus spinax***  (38/28) | ***Galeus melastomus***  (38/23) |  |  |
| --- | --- | --- | --- | --- | --- | --- |
| Chondrichthyans | *Chimaera* |  | 96 | 93 | *C. monstrosa* |  |
|  | *Etmopterus* | 43 |  | 59 | *E. spinax* |  |
|  | *Galeus* | 38 | 53 |  | *G. melastomus* |  |
| Crustacea | *Pandalus* | 23 | 30 | 18 | *A. propinqvus* | 6 |
|  |  |  |  |  | *D. bonnieri* | 5 |
|  |  |  |  |  | *P. borealis* | 85 |
|  |  |  |  |  | *P. montagui* | 4 |
| Osteichthyes | *Argentina* | 90 | 92 | 92 | ***A. sillus*** | 92 |
|  |  |  |  |  | *A. sphyraena* | 8 |
|  | *Gadiculus* | 54 | 71 | 10 | ***G. argentus*** |  |
|  | *Glyptoceophalus* | 43 | 63 | 56 | *G. cynoglossus* |  |
|  | *Lepidorhombus* | 26 | 22 | 37 | *L. boscii* |  |
|  |  |  |  |  | *L. whiffiagonis* | 14 |
|  | *Lophius* | 23 | 30 | 35 | *L. piscatorius* | 86 |
|  | *Maurolicus* | 15 | 22 | 24 | *M. muelleri* |  |
|  | *Merlangius* | 30 |  | 35 | *M. merlangus* |  |
|  | *Merluccius* | 30 | 20 | 40 | *M. merluccius* |  |
|  | *Micromesistius* | 78 | 78 | 56 | ***M. poutassou*** |  |
|  | *Molva* | 30 | 30 | 38 | *M. dypterygia* | 14 |
|  |  |  |  |  | *M. molva* | 86 |
|  | *Physics* | 35 | 47 | 49 | *P. blennoides* |  |
|  | *Trisopterus* | 92 | 95 | 95 | ***T. esmarkii*** | 91 |
|  |  |  |  |  | *T. luscus* | >1 |
|  |  |  |  |  | *T. minutus* | 8 |
