# Supplementary material for: Moving north: Warmer waters expand populations of deep-water cartilaginous fishes into Arctic waters
Source: PLoS One. 2026 Mar 5;21(3):e0343778. doi: 10.1371/journal.pone.0343778 (PMC12962514; doi:10.1371/journal.pone.0343778)
Supplement: Attachment — Submitted filename: PONE-D-25-46321REV.pdf [file pone.0343778.s014.pdf]

PLOS ONE

Moving North: Warmer waters expand populations of deep-water cartilaginous fishes into Arctic waters.

--Manuscript Draft--

**Manuscript Number:**	PONE-D-25-46321
**Article Type:**	Research Article
**Full Title:**	Moving North: Warmer waters expand populations of deep-water cartilaginous fishes into Arctic waters.
**Short Title:**	Moving North
**Corresponding Author:**	Romaric Jac<br>UQAR-ISMER: Universite du Quebec a Rimouski Institut des sciences des sciences de la mer de Rimouski<br>Rimouski, Québec CANADA
**Keywords:**	Chondrichthyans; sharks, chimaeras; Climate change; Community shift; Species distribution modelling; Transboundary conservation
**Abstract:**	Continental shelf and deep ocean ecosystems are increasingly exposed to anthropogenic pressures including commercial fishing and climate change related environmental stressors. Among the most vulnerable taxa are chondrichthyans due to their life histories with low reproductive output and therefore lower rebound potentials. In temperate regions, many chondrichthyan species are expected to undergo poleward distributional shifts in response to ocean warming. However, the extent and drivers of these shifts remain poorly understood, particularly in deep-water environments. This study aims to assess long-term trends in distribution and abundance for three cartilaginous fish species in the Northeast Atlantic Ocean: the rabbitfish (Chimaera monstrosa), the velvet-belly lanternshark (Etmopterus spinax), and the blackmouth catshark (Galeus melastomus). Focusing on the northern fringe of their distribution, 26 years (1995–2020) of standardised data from Norwegian scientific bottom trawl surveys were analysed using generalized additive models and GIS-based spatial mapping. The results indicate that all three species have undergone significant northward shifts in abundance over the past two decades, although the magnitude and rate varied among species. Several of their prey species exhibited similar latitudinal shifts, suggesting a potential trophic linkage in response to changing thermal regimes. These findings support the hypothesis that warming waters in northern latitudes are driving the poleward redistribution of deep-water chondrichthyans as they seek to remain within their thermal preference ranges. Understanding these spatial responses is critical for informing conservation strategies and future fisheries management in rapidly changing high-latitude marine ecosystems.
**Order of Authors:**	Romaric Jac
	Jon Albretsen
	Hannes Höffle
	Robert James Lennox
	Arved Staby
	Fabian Zimmermann
	Claudia Junge
**Additional Information:**	
**Question**	**Response**
**Financial Disclosure**<br><br>Enter a financial disclosure statement that describes the sources of funding for the	The author(s) received no specific funding for this work.

Powered by Editorial Manager® and ProduXion Manager® from Aries Systems Corporation

work included in this submission. Review the submission guidelines for detailed requirements. View published research articles from *PLOS ONE* for specific examples.<br><br>This statement is required for submission and **will appear in the published article** if the submission is accepted. Please make sure it is accurate.<br><br>**Funded studies**<br>Enter a statement with the following details:<br>• Initials of the authors who received each award<br>• Grant numbers awarded to each author<br>• The full name of each funder<br>• URL of each funder website<br>• Did the sponsors or funders play any role in the study design, data collection and analysis, decision to publish, or preparation of the manuscript?<br><br>Did you receive funding for this work?	
**Competing Interests**<br><br>Use the instructions below to enter a competing interest statement for this submission. On behalf of all authors, disclose any competing interests that could be perceived to bias this work—acknowledging all financial support and any other relevant financial or non-financial competing interests.<br><br>This statement is required for submission and **will appear in the published article** if the submission is accepted. Please make sure it is accurate and that any funding sources listed in your Funding Information later in the submission form are also declared in your Financial Disclosure statement.<br><br>View published research articles from *PLOS ONE* for specific examples.	The authors have declared that no competing interests exist.

**NO authors have competing interests**<br><br>Enter: *The authors have declared that no competing interests exist.*<br><br>**Authors with competing interests**<br><br>Enter competing interest details beginning with this statement:<br><br>*I have read the journal's policy and the authors of this manuscript have the following competing interests: [insert competing interests here]*<br><br>* typeset	
**Ethics Statement**<br><br>Enter an ethics statement for this submission. This statement is required if the study involved:<br><br>• Human participants<br>• Human specimens or tissue<br>• Vertebrate animals or cephalopods<br>• Vertebrate embryos or tissues<br>• Field research<br><br>Write "N/A" if the submission does not require an ethics statement.<br><br>General guidance is provided below. Consult the submission guidelines for detailed instructions. **Make sure that all information entered here is included in the Methods section of the manuscript.**	N/A

**Format for specific study types**<br><br>**Human Subject Research (involving human participants and/or tissue)**<br>• Give the name of the institutional review board or ethics committee that approved the study<br>• Include the approval number and/or a statement indicating approval of this research<br>• Indicate the form of consent obtained (written/oral) or the reason that consent was not obtained (e.g. the data were analyzed anonymously)<br><br>**Animal Research (involving vertebrate animals, embryos or tissues)**<br>• Provide the name of the Institutional Animal Care and Use Committee (IACUC) or other relevant ethics board that reviewed the study protocol, and indicate whether they approved this research or granted a formal waiver of ethical approval<br>• Include an approval number if one was obtained<br>• If the study involved *non-human primates*, add *additional details* about animal welfare and steps taken to ameliorate suffering<br>• If anesthesia, euthanasia, or any kind of animal sacrifice is part of the study, include briefly which substances and/or methods were applied<br><br>**Field Research**<br><br>Include the following details if this study involves the collection of plant, animal, or other materials from a natural setting:<br>• Field permit number<br>• Name of the institution or relevant body that granted permission	
**Data Availability**<br><br>Authors are required to make all data underlying the findings described fully available, without restriction, and from the time of publication. PLOS allows rare exceptions to address legal and ethical concerns. See the PLOS Data Policy and FAQ for detailed information.	Yes - all data are fully available without restriction

A Data Availability Statement describing where the data can be found is required at submission. Your answers to this question constitute the Data Availability Statement and **will be published in the article**, if accepted.<br><br>**Important:** Stating 'data available on request from the author' is not sufficient. If your data are only available upon request, select 'No' for the first question and explain your exceptional situation in the text box.<br><br>Do the authors confirm that all data underlying the findings described in their manuscript are fully available without restriction?	
**Describe where the data may be found in full sentences. If you are copying our sample text, replace any instances of XXX with the appropriate details.**<br><br>• If the data are **held or will be held in a public repository**, include URLs, accession numbers or DOIs. If this information will only be available after acceptance, indicate this by ticking the box below. For example: *All XXX files are available from the XXX database (accession number(s) XXX, XXX.).*<br>• If the data are all contained **within the manuscript and/or Supporting Information files**, enter the following: *All relevant data are within the manuscript and its Supporting Information files.*<br>• If neither of these applies but you are able to provide **details of access elsewhere**, with or without limitations, please do so. For example:<br><br>*Data cannot be shared publicly because of [XXX]. Data are available from the XXX Institutional Data Access / Ethics Committee (contact via XXX) for researchers who meet the criteria for access to confidential data.*<br><br>*The data underlying the results presented in the study are available from (include the name of the third party*	The Norwegian coastal survey data are published with the following DOI: 10.21335/NMDC-749719729 and can be accessed through the Norwegian Marine Data Centre (NMDC), here: https://doi.org/10.21335/NMDC-749719729. CTD-measurements from the Norwegian coastal locations off Bud, Eggum and Ingøy are available online from http://www.imr.no/forskning/forskningsdata/stasjoner/index.html in addition to other locations. Temperature and salinity data are available from the global ocean model: Copernicus Global 1/12° Oceanic and Sea Ice GLORYS12 (Copernicus Marine Environment Monitoring Service; https://marine.copernicus.eu).

Powered by Editorial Manager® and ProduXion Manager® from Aries Systems Corporation

and contact information or URL).<br>• This text is appropriate if the data are owned by a third party and authors do not have permission to share the data.<br><br>* typeset	
Additional data availability information:	Tick here if the URLs/accession numbers/DOIs will be available only after acceptance of the manuscript for publication so that we can ensure their inclusion before publication.

Powered by Editorial Manager® and ProduXion Manager® from Aries Systems Corporation

Manuscript

Click here to access/download;Manuscript;Manuscript_Moving_North.docx

Moving North: Warmer waters expand populations of deep-water cartilaginous fishes into Arctic waters.

Romaric Jac[1,2,3*], Jon Albretsen[1], Hannes Höffle[1], Robert J. Lennox[4,5], Arved Staby[1], Fabian Zimmermann[1], Claudia Junge[1*]

1. Institute of Marine Research (IMR), P.O.Box 1870 Nordnes, 5817 Bergen, Norway

2. Institut des sciences de la mer de Rimouski, Université du Québec à Rimouski, Rimouski, Quebec, Canada

3. UMR DECOD (Ecosystem Dynamics and Sustainability), L'Institut Agro, Ifremer, INRAE, Rennes, France

4. Department of Biology, Dalhousie University, Halifax, Nova Scotia, Canada

5. Ocean Tracking Network, Department of Biology, Dalhousie University, Halifax, Nova Scotia, Canada

* Corresponding author:

Romaric Jac, Institut des Sciences de la Mer de Rimouski (ISMER), 310 Allée des Ursulines, QC G5L 2Z9, Rimouski, Canada; romaric.jac@uqar.ca

Claudia Junge, IMR, P.O.Box 1870 Nordnes, 5817 Bergen, Norway; claudia.junge@hi.no

Abstract

Continental shelf and deep ocean ecosystems are increasingly exposed to anthropogenic pressures including commercial fishing and climate change related environmental stressors. Among the most vulnerable taxa are chondrichthyans due to their life histories with low reproductive output and therefore lower rebound potentials. In temperate regions, many chondrichthyan species are expected to undergo poleward distributional shifts in response to ocean warming. However, the extent and drivers of these shifts remain poorly understood, particularly in deep-water environments. This study aims to assess long-term trends in distribution and abundance for three cartilaginous fish species in the Northeast Atlantic Ocean: the rabbitfish (*Chimaera monstrosa*), the velvet-belly lanternshark (*Etmopterus spinax*), and the blackmouth catshark (*Galeus melastomus*). Focusing on the northern fringe of their distribution, 26 years (1995–2020) of standardised data from Norwegian scientific bottom trawl surveys were analysed using generalized additive models and GIS-based spatial mapping. The results indicate that all three species have undergone significant northward shifts in abundance over the past two decades, although the magnitude and rate varied among species. Several of their prey species exhibited similar latitudinal shifts, suggesting a potential trophic linkage in response to changing thermal regimes. These findings support the hypothesis that warming waters in northern latitudes are driving the poleward redistribution of deep-water chondrichthyans as they seek to remain within their thermal preference ranges. Understanding these spatial responses is critical for informing conservation strategies and future fisheries management in rapidly changing high-latitude marine ecosystems.

Keywords: Chondrichthyans; sharks, chimaeras; Climate change; Community shift; Species distribution modelling; Transboundary conservation

Introduction

Climate change is the main driver of contemporary ecological change [1] and is disrupting biological communities worldwide. Ocean warming directly and indirectly affects species distribution, growth, and mortality, with northern waters being most impacted by global warming [2]. In these waters, climate change is linked to 'Atlantification', a phenomenon closely related to the progression of anomalies from the Atlantic sector of the sub-Arctic seas into polar latitudes [3]. Predicting climate-driven impacts on biodiversity is challenging because biological responses are highly variable [4]. Nonetheless, evidence shows that climate change can alter community diversity and shift the abundance and distribution of mobile marine species [e.g., [5–13]. The magnitude of these impacts depends on the sensitivity (e.g., thermal tolerance), dispersal capacity (e.g., migratory *versus* non-migratory), and ability of the species to exploit new resources (generalist *versus* specialist feeder).

In the Northeast Atlantic Ocean, biomasses of chondrichthyans (sharks, rays, skates, chimaeras), have declined markedly in recent decades [14,15], with unknown and potentially disruptive ecological effects [16]. Climate-driven shifts in elasmobranch communities have already been documented in the North Sea, where several species exhibit changes in habitat suitability [7]. At the same time, growing recognition of the ecological importance of deep-water chondrichthyans [17,18] has prompted fishery managers to introduce measures in the Northeast Atlantic, such as total allowable catch reductions and recently direct fishing prohibitions, size, depth and gear restrictions, and partial habitat closures [e.g., [19,20]; NEAFC Rec Rec 08 2024; EU Reg. 2016/2336; EU Reg. 41/2007). Despite this, distributions of most chondrichthyans in the Norwegian Sea (defined by the International Council for the Exploration of the Sea, ICES, www.ices.dk) and the Barents Sea ecoregions (NSE and BSE, respectively) remain poorly defined, while bycatch rates continue to be high [14,21–24].

Understanding distribution patterns of temperate sharks and other chondrichthyans, particularly in deeper waters, is therefore essential for effective species and ecosystem management [25,26]. Yet research on deep-water taxa lags compared to their shallower counterparts. Here, we implemented species-specific models to examine long-term distributional changes in three common deep-water bycatch species across northern European waters: the rabbitfish *Chimaera monstrosa* Linnaeus, 1758, the velvet-belly lanternshark *Etmopterus spinax* (Linnaeus, 1758) and the blackmouth catshark *Galeus melastomus* Rafinesque, 1810. All inhabit broad depth ranges (104–1 600 m) in the Northeast Atlantic [27] but are also encountered on shallower continental shelves. *C. monstrosa* extends furthest north, to ~75° N (Dagit & Hareide, 2015; Guallart et al., 2015) and nearly 73° N off Norway [28]. Although *C. monstrosa* and *E. spinax* co-occur in Norwegian waters, differing diets and reproductive modes suggest limited competition [28].

Given the paucity of data for these species [27], this study provides a crucial step towards characterising their biogeography and ecology in the Northeast Atlantic. *Chimaera monstrosa* and *E. spinax* are listed as ‘Near Threatened’ and *G. melastomus* as ‘Least Concern’ on the IUCN Red List (Abella et al., 2015; Dagit & Hareide, 2015; Guallart et al., 2015), yet all three have recently been designated as Protected, Endangered or Threatened Species (PETS) by ICES in the NSE—and in the BSE for *C. monstrosa*. Existing knowledge of their contemporary distribution and habitat preferences [28,29] is insufficient to forecast responses to global change. This study therefore quantifies the influence of shifting environmental variables on these deep-water chondrichthyans at the fringe of their northern distribution range. Demonstrating changes in their biogeographic patterns across an extensive latitudinal range and multi-decadal timeline will help establish a baseline for long-term monitoring and adaptive management.

Materials and methods

Time series survey data

This study uses a 26-year (1995-2020) time series to investigate the impact of environmental variation on species distribution, drawing on an annual bottom trawl survey ("Kysttokt"; ICES-acronym: NOcoast-Aco/BTr-Q4) conducted along the Norwegian coast of the NSE and BSE. The dataset includes 18 years (2003-2020) of the "Kysttokt" data and 8 years (1995-2002) of bycatch records from the preceding combined saithe/coastal cod survey (Figure 1; details in [28]). In this study, the entire dataset—referred to as 'Kysttokt'—consists exclusively of scientific trawl data, comprising 3,159 hauls, each lasting 30 minutes at an average speed of 3.17 knots. A Campelen 1800 ma bottom trawl ("Shrimp trawl") with a 20 mm mesh size was used, suitable for quantitative capture of all three species, all of which are ≥10 cm at birth. The Norwegian Institute of Marine Research (IMR, Norway; and for the earlier years also Nofima, Norway) conducted these surveys between September and December each year. As a result, the data are representative of the distribution of these species during this period of the year. Most bottom trawl stations were fixed and on average, 122 stations were trawled per year. Despite some nonsignificant changes, the spatiotemporal coverage of hauls can be considered homogeneous, thereby minimising the capture bias (Figures S1 and S2 and Table S1).

As fjord water masses differ environmentally from coastal and offshore waters, stations were classified as either 'Outside' or 'Inside' fjord, using the Norwegian Environment Agency’s fjord catalogue (https://www.environmentagency.no/). 'Inside Fjord' stations were removed from the

dataset. Abundance was standardised following [28], using trawl distance and a mean trawl opening width of 25 m to calculate density (individuals per km^2).

Environmental and spatial data

Following [28], five environmental and spatial covariates were used to explain species presence: maximum trawl depth, near-bottom temperature, near-bottom salinity, distance from the coast, and latitude. Distance to the coast (in km) was calculated using the Near Neighbour Join tool in QGIS. To obtain reliable temperature and salinity data that best reflect conditions throughout the study area since 1995, monthly average temperature and salinity data at every 10m depths were extracted from a global ocean model: Copernicus Global 1/12° Oceanic and Sea Ice GLORYS12 (Copernicus Marine Environment Monitoring Service; https://marine.copernicus.eu). This three-dimensional model includes sea ice dynamics and has a horizontal resolution of 1/12° (9.25 km at the equator and around 4.5 km at subpolar latitudes) and a vertical grid with a resolution of 1 m near the surface to 450 m below 5000 m depth [30]. Monthly averages of salinity and temperature were then associated with each trawl station location (i.e., average latitude, longitude and depth).

Although GLORYS is widely validated, we specifically compared its estimates against CTD data from three IMR stations (Bud at 63°N, Eggum at 68°N, and Ingøy at 71°N) between 1993 and 2020 for this study. The evaluation confirmed that it reliably reproduces hydrographical properties across all depths and supports the GLORYS model's suitability for use in this study area, accurately representing the environmental conditions of our region. Details are found in the Supplementary text and Figures S3 and S4.

Analysis of species habitats

Habitat occupancy (extent of habitat use) and preference (most suitable or favoured habitat) were assessed for each species using the full catch dataset. Habitat was characterised using five environmental and spatial variables identified in advance as important predictors of demersal chondrichthyan distribution [15,28,31]. Habitat preference was determined by identifying the environmental conditions associated with the top 70% of individual captures, representing the most frequently used habitat conditions.

Changes in abundance and distribution

To evaluate changes in species' abundance and distribution over a 26-year period, three approaches were employed: (1) Generalised Additive Models (GAMs) to analyse spatial distribution; (2) Mapping habitat occupancy at the start (1995) and end (2020) of the time series; (3) Tracking changes in centre of gravity (COG) over time using depth, latitude, and distance from the coast.

Model development, selection, and evaluation

GAMs, an extension of generalised linear models, allow inclusion of smoothed terms (splines) to capture non-linear relationships [32,33]. The models were fitted using the 'mgcv' package in R [24,34], with a negative binomial distribution for catch count data. Predictor smooths were chosen through data exploration and model selection.

Collinearity among predictor variables was tested prior to modelling. Highly correlated variables (Pearson's $r > 0.7$) were excluded [35], as well as variables with variance inflation factor (VIF) values >2.5 [36]. No pairwise correlations (Figure S5) and multicollinearity among the predictor variables were found.

A forward selection process based on Akaike Information Criterion (AIC; [37]) was used to identify the most parsimonious model. ANOVA tests compared simpler and more complex models at each step. Final models were assessed using AIC, adjusted R^2, and explained deviance.

Mapping the distribution change

To quantify spatial change over time, habitat occupancy maps were generated for 1995 and 2020 using QGIS. Change was measured as the proportion of non-overlapping areas. The COG for each year was calculated for the three spatial variables, weighting trawl locations by standardised species abundance (approach in [5]). Linear regressions were then used to estimate shifts in species' mean location and depth between 1995 and 2020.

Temporal changes of potential prey assemblage

To assess possible trophic interactions, binary presence–absence data from the Kysttokt survey were used to evaluate co-occurrence between the three focal chondrichthyans and other species. A species was considered a potential prey if it (1) had a high probability of positive co-occurrence, (2) was morphologically and size suitable as prey and (3) had supporting evidence from other regions. Using

the ‘cooccur’ R package [38], yearly and latitudinal co-occurrence patterns were classified as positive, negative, or random. Co-occurrence frequency was expressed as the percentage of shared hauls relative to the total. Non-random associations may indicate trophic or ecological interactions. Distributional trends of positively associated potential prey species were analysed over time.

Results

Time series of bottom water temperatures and salinities

The temperature shows an increasing trend from the 1990s, with an absolute error below one unit (°C/psu) throughout all depths, seasons, and years (Figures S6-S9). The comprehensive temporal coverage (1995–2020), combined with low error margins, validates the use of these data as physical predictors.

Autumn near-bottom water temperatures increased between 1995 and 2016, followed by a slight decline to 2020 at the northernmost latitude (71°N). No significant temperature differences were found between shallow and deep stations. Waters north of 64°N showed a marked warming trend over time. The temperature–depth profiles followed expected patterns for the Northeast Atlantic. In contrast, near-bottom salinity remained relatively stable, between 34.1 and 35.7 for 95% of the dataset, with no significant temporal changes detected.

Species habitats occupancy and preference

The characteristics of habitat occupancy and preferences for the three species during the past 26 years are shown in Figure 2 and Table S3. *Chimaera monstrosa* exhibited the broadest habitat tolerance, occupying a wide range of latitude and, depths (including the maximum depth surveyed), and a broad temperature range (4.1 - 12.4°C). In contrast, *E. spinax* was found within a more limited geographical extent, associated with cooler waters (up to 10.5 °C). *Galeus melastomus* showed a similarly restricted geographical distribution, with a narrower preferred temperature range (7.2–9.1 °C) and occurring predominantly between 62.7° and 66.4°N. Salinity preferences were narrow across all three species, reflecting the relative homogeneity of near-bottom salinity in the study region rather than specific selection for different salinity conditions.

Changes in abundance and distribution

The negative binomial abundance model for NSE and BSE identified latitude as the only significant variable across all three species (Table 1). Moreover, for *C. monstrosa*, distance from the coast was also a significant variable (Figure S10).

Regarding the changes in latitude, over the 26-year period, a northward shift in latitudinal distribution and abundance was evident for all species, with associated changes in habitat occupancy: +26% for *C. monstrosa*, +63% for *E. spinax*, and +55% for *G. melastomus* (Figure 3a). Latitudinal COG time series confirmed these trends. *Chimaera monstrosa* and *E. spinax* each shifted more than +1°N—approximately 120 km northward—since the beginning of the Kysttokt survey, while *G. melastomus* shifted just under +1°N (Figure 3b). For *C. monstrosa*, the COG crossed the Arctic Circle in 1999 and 2001, and since the 2010s, it has consistently remained above this threshold. The years 2011 and 2017 mark the highest recorded latitudinal values. *Etmopterus spinax* remained mostly south of 66°N, with exceptions in 2013 and 2017. *G. melastomus* showed more stable interannual variation, with a general northward shift of ~70 km over the study period. No significant trends were found in the depth or coastal distance COG for any species.

Temporal changes of potential prey assemblage

The co-occurrence analysis included 191 species collected during the Kysttokt survey, representing 142 genera. These comprised 86 Osteichthyes, 18 Crustacea, 11 Cephalopoda, 10 Chondrichthyes, 8 Cnidaria, and 3 Mollusca. Among these species, 24 species co-occurred with at least one of the three focal chondrichthyan species at >1% frequency between 1995 and 2020 (Table 3).

Chimaera monstrosa exhibited the second highest rate of positive co-occurrences among all species, following *Micromesistius poutassou* (blue whiting). However, due to limited sampling of epibenthic fauna and small crustaceans in the Kysttokt survey, which are important components of *C. monstrosa*'s diet, no definitive conclusions could be drawn regarding its prey composition. For the two shark species, strong positive co-occurrence patterns were observed with *Trisopterus esmarkii* (Norway pout), *Argentina silus* (greater argentine), *M. poutassou*, *Gadiculus argenteus* (silvery pout), and *Glyptocephalus cynoglossus* (witch flounder). These associations are consistent with previous studies that reported trophic links such as predation by *E. spinax* on *M. poutassou* and *G. argenteus*, and by *G. melastomus* on *Argentina* spp. and *T. esmarkii* (Mattson, 1981; Bergstad et al., 2003; Besnard et al., 2022). Further analyses were conducted on these four prey species to assess shifts in their spatial distribution.

Both *G. argenteus* and *T. esmarkii* exhibited significant northward shifts, moving 1.7 and 3 degrees of latitude respectively (Figure 4) during the 26-year period. In contrast, *A. silus* and *M. poutassou* did not show any significant changes in latitude. As a result of these shifts, all potential prey species are now predominantly found between 64° and 70°N, with their centres of gravity located largely north of the Arctic Circle.

Discussion

Over the past 26 years, near-bottom water temperatures in the studied area have increased by approximately 1°C (Figures S5 and S6). These results align with previous research highlighting significant warming across the northern European continental shelf, particularly along the Norwegian coast [39], and record-breaking temperatures in the Barents Sea [40]. This warming is primarily attributed to the heat transport from lower latitudes via the Atlantic Meridional Overturning Circulation (AMOC), major current system regulating climate in the North Atlantic. Long-term AMOC records display large oscillations, with periods of strengthening and weakening observed during the study period [41,42], which have significantly impacted thermal conditions in northern seas.

Expanding Nordic shark and chimaera populations

This study reveals a northward shift of the COG of *C. monstrosa*, *E. spinax*, and *G. melastomus* by 42, 44 and 24 km per decade, respectively, likely in response to warming waters in the NSE and BSE. These findings contribute important evidence to a growing body of literature documenting poleward shifts in fishes [e.g., [5–13], similar to trends seen along the Northwest Atlantic coast [e.g., [10,43,44]. As chondrichthyans are particularly sensitive to temperature, and commonly migrate to maintain their optimal thermal niche [45]. The observed expansions reflect a broader borealisation of Arctic waters in response to Atlantification [3,46], with all three species now established within the NSE and BSE.

Species sensitivity to warming depends on thermal tolerance and capacity for acclimation [47]. The northward movements were non-linear and coincided with extreme temperature anomalies, especially during marine heatwaves (MHWs) defined by [48] as prolonged periods of anomalously warm water. MHWs have allowed long-term range extensions into more northern and offshore waters [49], particularly in deep coastal and fjord habitats that are thermally sensitive [50]. The frequency and intensity of MHWs have risen over the past century [51] and are projected to increase further [52]. Without a slowdown of the AMOC, Norwegian waters could reach near-permanent MHW conditions by late century [53]. Such changes may rapidly restructure populations, alter abundance

and shift habitat limits [54] and could influence growth rates [55]. Conversely, a mid-century AMOC collapse, as some scenarios suggest, would likely intensify climate variability and accelerate ecosystem change [56].

Stable abundances in southern survey areas despite northward movements suggest either range expansion or a simultaneous southern contraction lying outside our sampling domain. Migrants moving north may be replaced by individuals from regions farther south such as the Skagerrak, Celtic Sea, Faroes or Iceland where *C. monstrosa* and *E. spinax* also occur. The absence of *Chimaera opalescens* and other chimaeriforms in Norwegian waters, despite their presence farther south (Jakobsdóttir et al., 2020), hints at a biogeographical barrier that limits northward dispersal. Although no basin-wide study has been conducted, distributional changes for all three species have been reported in the Bay of Biscay [57], supporting large-scale redistribution of deep-water chondrichthyans under warming scenarios.

Feeding ecology and prey dynamics

Predator distributions are closely linked to ecological interactions such as predation, competition, and facilitation [58]. Rising temperatures are expected to reorganise marine food webs [59], leading to alterations in predator ranges [60,61]. Two of the four identified prey species, *G. argenteus* and *T. esmarkii*, have shifted significantly northward. *Micromesistius poutassou* has also extended into the northern and eastern Barents Sea, taking advantage of warmer water and reduced sea ice that enhance primary production [62]. Although our analysis centred on prey availability through co-occurrence, substrate type might influence prey habitat. Because benthoscape data were coarse, substrate was not included in our models but should be examined when finer-scale information becomes available.

Prey now reside mostly above the Arctic Circle, creating only limited overlap with the range of *G. melastomus*. Continued divergence could deprive this shark of key prey resources. Both shark species also feed on crustaceans; *E. spinax* targets *Meganyctiphanes norvegica* [63] and *G. melastomus* targets *Pandalus borealis* [64]. These crustaceans remain widespread in the Barents Sea [65,66]. A switch to crustaceans could buffer sharks against fish prey loss, as seen in other species affected by warming [67,68]. However, increased reliance on *P. borealis* may raise bycatch risk because deep-water shrimp fisheries capture many chondrichthyans [69]. For *C. monstrosa*, a highly specialised diet that in some areas consists almost exclusively of *Monodaeus couchii* [70] could limit resilience to climate change [71,72].

Study limitation and broader implication

While this study focused on the influence of warming on species distribution and abundance, a broader suite of climate-related stressors could also shape ecological dynamics in northern marine ecosystems. Factors such as sea ice decline, changes in light availability, nutrient fluxes, ocean pollution, acidification, deoxygenation, and shifting current patterns can each drive structural and functional changes in marine communities [73,74]. Additionally, oceanographic variation across different basins may mediate species' responses to climate change in distinct ways [71]. Altered hydrographic conditions can also lead to the relocation of spawning grounds and push fishing activities further north or into deeper waters [75], though such dynamics were beyond the scope of this study. A further limitation is the temporal window of our dataset: surveys were conducted only between September and December. Although no pronounced seasonal patterns have been reported for the focal species to date, restricting observations to autumn may overlook potential intra-annual shifts in distribution and habitat use that occur at other times of the year. Expanding sampling to additional seasons would help determine whether the patterns documented here persist year-round or vary seasonally.

Given the forecasted intensification of Atlantification and extreme climatic events, greater research attention should be directed toward deep-water species at the edges of their distribution ranges. These species are often poorly understood but may serve as important indicators of ecological change. Range shifts are already reshaping species assemblages, with potential ripple effects for ecosystem functioning and human livelihoods [76]. Monitoring and managing new species arriving in Arctic and sub-Arctic waters will be increasingly complex and must account not only for expanding range fronts but also for contractions at the trailing (cold) edges of distributions [77]. In this study, all three examined cartilaginous species are expanding their ranges and adjusting their abundance to remain within favourable thermal conditions in the Norwegian and Barents Sea regions. If current trends persist, further expansion into higher latitudes such as the White Sea is plausible. Ensuring the sustainable management of these transboundary species, particularly in the Barents Sea shared by Norway and Russia, will require strengthened international collaboration, joint monitoring frameworks, and open exchange of data across national boundaries.

Acknowledgements

We would like to thank cruise leaders, research scientists and technicians at the Institute of Marine Research (IMR) in Norway for providing the survey data, Caroline A. Tranang for help with the data preparation, as well as Laurène Merillet and Benjamin Planque for ideas for data analysis and for constructive discussions, and the research group on Deepwater and cartilaginous fishes for their support and helpful discussions. Thanks to the IMR for funding RJ to publish his MSc thesis results.

Data availability

The Norwegian coastal survey data are published with the following DOI: 10.21335/NMDC-749719729 and can be accessed through the Norwegian Marine Data Centre (NMDC), here: https://doi.org/10.21335/NMDC-749719729.

CTD-measurements from the Norwegian coastal locations off Bud, Eggum and Ingøy are available online from http://www.imr.no/forskning/forskningsdata/stasjoner/index.html in addition to other locations. Temperature and salinity data are available from the global ocean model: Copernicus Global 1/12° Oceanic and Sea Ice GLORYS12 (Copernicus Marine Environment Monitoring Service; https://marine.copernicus.eu).

Funding

IMR provided funding through the Norwegian Sea programme for RJ. IMR conducted the scientific surveys to collect the data used here, and financed JA, HH, AS, FZ and CJ.

Conflict of interest disclosure

The authors have declared that no competing interests exist.

Author contributions

Conceptual idea (CJ, RJ), data generation (CJ, JA, AS, HH), data analysis (RJ, JA), model development (RJ, RJL, HH, FZ), validation (CJ, JA, HH, RJL, FZ), supervision (CJ), discussion of results (all), manuscript preparation (RJ, CJ, with contributions from all authors).

References

1. Rubenstein MA, Weiskopf SR, Carter SL, Eaton MJ, Johnson C, Lynch AJ, et al. Do empirical observations support commonly-held climate change range shift hypotheses? A systematic review protocol. Environ Evid. 2020;9: 1–10. doi:10.1186/s13750-020-00194-9

2. Bentley JW, Serpetti N, Heymans JJ. Investigating the potential impacts of ocean warming on the Norwegian and Barents Seas ecosystem using a time-dynamic food-web model. Ecol Modell. 2017;360: 94–107. doi:10.1016/j.ecolmodel.2017.07.002

3. Ingvaldsen RB, Assmann KM, Primicerio R, Fossheim M, Polyakov I V., Dolgov A V. Physical manifestations and ecological implications of Arctic Atlantification. Nat Rev Earth Environ. 2021;2: 874–889. doi:10.1038/s43017-021-00228-x

4. Lenoir J, Gégout JC, Guisan A, Vittoz P, Wohlgemuth T, Zimmermann NE, et al. Going against the flow: potential mechanisms for unexpected downslope range shifts in a warming climate. Ecography. 2010;33: 295–303. doi:10.1111/j.1600-0587.2010.06279.x

5. Campana SE, Stefánsdóttir RB, Jakobsdóttir K, Sólmundsson J. Shifting fish distributions in warming sub-Arctic oceans. Sci Rep. 2020;10: 1–14. doi:10.1038/s41598-020-73444-y

6. Coulon N, Lindegren M, Goberville E, Toussaint A, Receveur A, Auber A. Threatened fish species in the Northeast Atlantic are functionally rare. Global Ecology and Biogeography. 2023;32: 1827–1845. doi:10.1111/geb.13731

7. Coulon N, Elliott S, Teichert N, Auber A, McLean M, Barreau T, et al. Northeast Atlantic elasmobranch community on the move: functional reorganization in response to climate change. Glob Chang Biol. 2024;30: e17157. doi:10.1111/gcb.17157

8. Cheung WWL, Watson R, Pauly D. Signature of ocean warming in global fisheries catch. Nature. 2013;497: 365–368. doi:10.1038/nature12156

9. Dulvy NK, Rogers SI, Jennings S, Stelzenmüller V, Dye SR, Skjoldal HR. Climate change and deepening of the North Sea fish assemblage: a biotic indicator of warming seas. Journal of Applied Ecology. 2008;45: 1029–1039. doi:10.1111/j.1365-2664.2008.01488.x

10. Gordó-Vilaseca C, Stephenson F, Coll M, Lavin C, Costello MJ. Three decades of increasing fish biodiversity across the northeast Atlantic and the Arctic Ocean. Proceedings of the National Academy of Sciences. 2023;120: e2120869120. doi:10.1073/pnas.2120869120

11. Rutterford LA, Simpson SD, Bogstad B, Devine JA, Genner MJ. Sea temperature is the primary driver of recent and predicted fish community structure across Northeast Atlantic shelf seas. Glob Chang Biol. 2023. doi:10.1111/gcb.16633

12. Simpson SD, Jennings S, Johnson MP, Blanchard JL, Schön PJ, Sims DW, et al. Continental shelf-wide response of a fish assemblage to rapid warming of the sea. Current Biology. 2011;21: 1565–1570. doi:10.1016/j.cub.2011.08.016

13. Siwertsson A, Lindström U, Aune M, Berg E, Skarðhamar J, Varpe Ø, et al. Rapid climate change increases diversity and homogenizes composition of coastal fish at high latitudes. Glob Chang Biol. 2024;30: e17273. doi:10.1111/gcb.17273

14. ICES. Report of the Working Group on Elasmobranch Fishes (WGEF). ICES Scientific Reports. 2024;06: 994. doi:10.17895/ices.pub.26935504

15. Sguotti C, Lynam CP, García-Carreras B, Ellis JR, Engelhard GH. Distribution of skates and sharks in the North Sea: 112 years of change. Glob Chang Biol. 2016;22: 2729–2743. doi:10.1111/gcb.13316

16. Benn AR, Weaver PP, Billet DSM, van den Hove S, Murdock AP, Doneghan GB, et al. Human activities on the deep seafloor in the North East Atlantic: an assessment of spatial extent. PLoS One. 2010;5: 1–15. doi:10.1371/journal.pone.0012730

17. Hammerschlag N, Schmitz OJ, Flecker AS, Lafferty KD, Sih A, Atwood TB, et al. Ecosystem function and services of aquatic predators in the anthropocene. Trends Ecol Evol. 2019;34: 369–383. doi:10.1016/j.tree.2019.01.005

18. Shiffman DS, Ajemian MJ, Carrier JC, Daly-Enge TS, Davis MM, Dulvy NK, et al. Trends in chondrichthyan research: an analysis of three decades of conference abstracts. Copeia. 2020;108: 122–131. doi:10.1643/OT-19-179R

19. Ellis JR, Clarke MW, Cortés E, Heessen HJL, Apostolaki P, Carlson JK, et al. Management of elasmobranch fisheries in the North Atlantic. Advances in Fisheries Science: 50 years on from Beverton and Holt. Blackwell Publishing Oxford; 2008. pp. 184–228.

20. Pawson MG, Ellis JR. Stock identity of elasmobranchs in the Northeast Atlantic in relation to assessment and management. Journal of Northwest Atlantic Fishery Science. 2005;35: 173–193. doi:10.2960/J.v35.m480

21. ICES. Barents Sea Ecosystem – Fisheries overview. In Report of the ICES Advisory Committee, 2021 ICES Advice: Fisheries Overviews. 2021; 29. doi:10.17895/ices.advice.9166

22. ICES. Norwegian Sea ecoregion – Fisheries Overview. In Report of the In Report of the ICES Advisory Committee, 2022 ICES Advice 2022, section 122. 2022; 29. doi:10.17895/ices.advice.21640826

23. Finucci B, Pacoureau N, Rigby CL, Matsushiba JH, Faure-Beaulieu N, Sherman CS, et al. Fishing for oil and meat drives irreversible defaunation of deepwater sharks and rays. Science (1979). 2024;383: 1135–1141. doi:10.1126/science.ade9121

24. Howell KL, Hilário A, Allcock AL, Bailey D, Baker M, Clark MR, et al. A decade to study deep-sea life. Nat Ecol Evol. 2021;5: 265–267. doi:10.1038/s41559-020-01352-5

25. Espinoza M, Araya-Arce T, Chaves-Zamora I, Chinchilla I, Cambra M. Monitoring elasmobranch assemblages in a data-poor country from the Eastern Tropical Pacific using baited remote underwater video stations. Sci Rep. 2020;10: 1–18. doi:10.1038/s41598-020-74282-8

26. Gouraguine A, Hidalgo M, Moranta J, Bailey DM, Ordines F, Guijarro B, et al. Elasmobranch spatial segregation in the western Mediterranean. Sci Mar. 2011;75: 653–664. doi:10.3989/scimar.2011.75n4653

27. ICES. Workshop on the distribution and bycatch management options of listed deep-sea shark species (WKSHARK6). ICES Scientific Reports. 2020;2: 85. doi:10.17895/ices.pub.7469

28. Jac R, Höffle H, Albretsen J, Jakobsdóttir K, Staby A, Søvik G, et al. Of three sharks and one chimaera: varied habitat preferences across a latitudinal range revealed by coastal and offshore surveys. J Fish Biol. 2022;100: 660–674. doi:10.1111/jfb.14979

29. Mattína H, Campana SE, Jakobsdóttir K. Environmental preferences and critical habitat for the velvet belly lanternshark (*Etmopterus spinax*) in Icelandic waters. PLoS One. 2024;19: e0299544-. doi:10.1371/journal.pone.0299544

30. Lellouche J-M, Greiner E, Bourdallé Badie R, Garric G, Melet A, Drévillon M, et al. The Copernicus Global 1/12° Oceanic and Sea Ice GLORYS12 reanalysis. Front Earth Sci (Lausanne). 2021;9: 1–27. doi:10.3389/feart.2021.698876

31. Martin CS, Vaz S, Ellis JR, Lauria V, Coppin F, Carpentier A. Modelled distributions of ten demersal elasmobranchs of the eastern English Channel in relation to the environment. J Exp Mar Biol Ecol. 2012;418: 91–103. doi:10.1016/j.jembe.2012.03.010

32. Hastie T, Tibshirani R. Exploring the nature of covariate effects in the proportional hazards model. International Biometric Society. 1990;46: 1005–1016. doi:10.2307/2532444

33. Wood SN. On confidence intervals for generalized additive models based on penalized regression splines. Aust N Z J Stat. 2006;48: 445–464. doi:10.1111/j.1467-842X.2006.00450.x

34. RStudio. RStudio: Integrated Development for R. RStudio, PBC, Boston, MA URL; 2020. Available: http://www.rstudio.com/

35. Dormann CF, Elith J, Bacher S, Buchmann C, Carl G, Carré G, et al. Collinearity: a review of methods to deal with it and a simulation study evaluating their performance. Ecography. 2013;36: 27–46. doi:10.1111/j.1600-0587.2012.07348.x

36. Johnston R, Jones K, Manley D. Confounding and collinearity in regression analysis: a cautionary tale and an alternative procedure, illustrated by studies of British voting behaviour. Qual Quant. 2018;52: 1957–1976. doi:10.1007/s11135-017-0584-6

37. Akaike H. A new look at the statistical model identification. IEEE Trans Automat Contr. 1974;19: 716–723. doi:10.1109/TAC.1974.1100705

38. Griffith DM, Veech JA, Marsh CJ. Cooccur: probabilistic species co-occurrence analysis in R. J Stat Softw. 2016;69: 1–17. doi:10.18637/jss.v069.c02

39. González-Pola C, Larsen K, Fratantoni P, Beszczynska-Moller A. ICES Report on Ocean Climate 2018. ICES Cooperative Research Report. 2019; 122pp. doi:10.17895/ices.pub.5461

40. Smedsrud LH, Esau I, Ingvaldsen RB, Eldevik T, Haugan PM, Li C, et al. The role of the Barents Sea in the Arctic climate system. Reviews of Geophysics. 2013;51: 415–449. doi:10.1002/rog.20017

41. Jackson LC, Biastoch A, Buckley MW, Desbruyères DG, Frajka-Williams E, Moat B, et al. The evolution of the North Atlantic meridional overturning circulation since 1980. Nat Rev Earth Environ. 2022;3: 241–254. Available: https://doi.org/10.1038/s43017-022-00263-2

42. Trenberth KE, Zhang Y, Fasullo JT, Cheng L. Observation-based estimates of global and basin ocean meridional heat transport time series. J Clim. 2019;32: 4567–4583. doi:10.1175/JCLI-D-18

43. Adams CF, Alade LA, Legault CM, O'Brien L, Palmer MC, Sosebee KA, et al. Relative importance of population size, fishing pressure and temperature on the spatial distribution of nine Northwest Atlantic groundfish stocks. PLoS One. 2018;13: e0196583. doi:10.1371/journal.pone.0196583

44. Frainer A, Primicerio R, Kortsch S, Aune M, Dolgov A V., Fossheim M, et al. Climate-driven changes in functional biogeography of Arctic marine fish communities. Proc Natl Acad Sci U S A. 2017;114: 12202–12207. doi:10.1073/pnas.1706080114

45. Schlaff AM, Heupel MR, Simpfendorfer CA. Influence of environmental factors on shark and ray movement, behaviour and habitat use: a review. Rev Fish Biol Fish. 2014;24: 1089–1103. doi:10.1007/s11160-014-9364-8

46. Fossheim M, Primicerio R, Johannesen E, Ingvaldsen RB, Aschan MM, Dolgov A V. Recent warming leads to a rapid borealization of fish communities in the Arctic. Nat Clim Chang. 2015;5: 673–677. doi:10.1038/nclimate2647

47. Rodriguez-Dominguez A, Connell SD, Leung JYS, Nagelkerken I. Adaptive responses of fishes to climate change: feedback between physiology and behaviour. Science of the Total Environment. 2019;692: 1242–1249. doi:10.1016/j.scitotenv.2019.07.226

48. Hobday AJ, Alexander L V., Perkins SE, Smale DA, Straub SC, Oliver ECJ, et al. A hierarchical approach to defining marine heatwaves. Prog Oceanogr. 2016;141: 227–238. doi:10.1016/j.pocean.2015.12.014

49. Smale DA, Wernberg T, Oliver ECJ, Thomsen M, Harvey BP, Straub SC, et al. Marine heatwaves threaten global biodiversity and the provision of ecosystem services. Nat Clim Chang. 2019;9: 306–312. doi:10.1038/s41558-019-0412-1

50. Borgman E, Pedersen MF, Upadhyay Stæhr PA, Fischer-Bogason R. Marine heatwaves in Northen Sea areas: occurrence, effects, and expected frequencies. PlanMiljø. PlanMiljø. 2022; 38.

51. Oliver ECJ, Donat MG, Burrows MT, Moore PJ, Smale DA, Alexander L V., et al. Longer and more frequent marine heatwaves over the past century. Nat Commun. 2018;9: 1–12. doi:10.1038/s41467-018-03732-9

52. Frölicher TL, Fischer EM, Gruber N. Marine heatwaves under global warming. Nature. 2018;560: 360–364. doi:10.1038/s41586-018-0383-9

53. Ren X, Liu W. The role of a weakened Atlantic meridional overturning circulation in modulating marine heatwaves in a warming climate. Geophys Res Lett. 2021;48: e2021GL095941. doi:10.1029/2021GL095941

54. Harvey BP, Marshall KE, Harley CDG, Russell BD. Predicting responses to marine heatwaves using functional traits. Trends in Ecology and Evolution. Elsevier Ltd; 2022. pp. 20–29. doi:10.1016/j.tree.2021.09.003

55. Vollset KW, Urdal K, Utne K, Thorstad EB, Saegrov H, Raunsgard A, et al. Ecological regime shift in the Northeast Atlantic Ocean revealed from the unprecedented reduction in marine growth of Atlantic salmon. Sci Adv. 2022;8: eabk2542. Available: https://www.science.org/doi/pdf/10.1126/sciadv.abk2542

56. Ditlevsen P, Ditlevsen S. Warning of a forthcoming collapse of the Atlantic meridional overturning circulation. Nat Commun. 2023;14. doi:10.1038/s41467-023-39810-w

57. Punzón A, Serrano A, Sánchez F, Velasco F, Preciado I, González-Irusta JM, et al. Response of a temperate demersal fish community to global warming. Journal of Marine Systems. 2016;161: 1–10. doi:10.1016/j.jmarsys.2016.05.001

58. Gervais CR, Huveneers C, Rummer JL, Brown C. Population variation in the thermal response to climate change reveals differing sensitivity in a benthic shark. Glob Chang Biol. 2021;27: 108–120. doi:10.1111/gcb.15422

59. Wernberg T, Russell BD, Moore PJ, Ling SD, Smale DA, Campbell A, et al. Impacts of climate change in a global hotspot for temperate marine biodiversity and ocean warming. Journal of Experimental Marine Biology and Ecology. 2011. pp. 7–16. doi:10.1016/j.jembe.2011.02.021

60. Lurgi M, López BC, Montoya JM. Novel communities from climate change. Philosophical Transactions of the Royal Society B: Biological Sciences. 2012;367: 2913–2922. doi:10.1098/rstb.2012.0238

61. Cameron EK, Sundqvist MK, Keith SA, CaraDonna PJ, Mousing EA, Nilsson KA, et al. Uneven global distribution of food web studies under climate change. Ecosphere. 2019;10: e02645. doi:10.1002/ecs2.2645

62. Hátún H, Payne MR, Beaugrand G, Reid PC, Sandø AB, Drange H, et al. Large bio-geographical shifts in the north-eastern Atlantic Ocean: From the subpolar gyre, via plankton, to blue whiting and pilot whales. Prog Oceanogr. 2009;80: 149–162. doi:10.1016/j.pocean.2009.03.001

63. Besnard L, Duchatelet L, Bird CS, Le Croizier G, Michel L, Pinte N, et al. Diet consistency but large-scale isotopic variations in a deep-sea shark: The case of the velvet belly lantern shark, *Etmopterus spinax*, in the northeastern Atlantic region and Mediterranean Sea. Deep Sea Research Part I: Oceanographic Research Papers. 2022;182: 103708. doi:10.1016/j.dsr.2022.103708

64. Mattson S. The food of *Galeus melastomus*, *Gadiculus argenteus thori*, *Trisopterus esmarkii*, *Rhinonemus cimbrius*, and *Glyptocephalus ctnoglossus* (pisces) caught during the day with shrimp trawl in a West-Norwegian fjord. Sarsia. 1981;66: 109–127. doi:10.1080/00364827.1981.10414529

65. Dalpadado P, Hop H, Rønning J, Pavlov V, Sperfeld E, Buchholz F, et al. Distribution and abundance of euphausiids and pelagic amphipods in Kongsfjorden, Isfjorden and Rijpfjorden (Svalbard) and changes in their relative importance as key prey in a warming marine ecosystem. Polar Biol. 2016;39: 1765–1784. doi:10.1007/s00300-015-1874-x

66. Garcia EG. The northern shrimp (*Pandalus borealis*) offshore fishery in the Northeast Atlantic. Adv Mar Biol. 2007;52: 147–266. doi:10.1016/S0065-2881(06)52002-4

67. Freitas C, Olsen EM, Moland E, Ciannelli L, Knutsen H. Behavioral responses of Atlantic cod to sea temperature changes. Ecol Evol. 2015;5: 2070–2083. doi:10.1002/ece3.1496

68. Townhill BL, Holt RE, Bogstad B, Durant J l. M, Pinnegar JK, Dolgov A V., et al. Diets of the Barents Sea cod (*Gadus morhua*) from the 1930s to 2018. Earth Syst Sci Data. 2021;13: 1361–1370. doi:10.5194/essd-13-1361-2021

69. Börjesson P, Norén K, Valentinsson D. Occurrence of sharks, rays and rabbit fish in the Greater North Sea – and catches in Swedish fisheries. Department of Aquatic Resources, Sveriges lantbruksuniversitet Aqua reports; 2022:14. 2022; 41. Available: https://res.slu.se/id/publ/116702

70. Tamayo M, Barría C, Coll M, Navarro J. Highly specialized feeding habits of the rabbitfish *Chimaera monstrosa* in the deep sea ecosystem of the northwestern Mediterranean Sea. Journal of Applied Ichthyology. 2021;37: 868–874. doi:10.1111/jai.14241

71. Hoegh-Guldberg O, Bruno JF. The impact of climate change on the world's marine ecosytems. Science (1979). 2010;328: 1523–1528. Available: https://www.science.org/doi/full/10.1126/science.1189930

72. Pinsky ML, Worm B, Fogarty M, Sarmiento JL, Levin SA. Marine taxa track local climate velocities. Science (1979). 2013;341: 1239–1242. doi:10.1126/science.1239352

73. Dalpadado P, Ingvaldsen RB, Stige LC, Bogstad B, Knutsen T, Ottersen G, et al. Climate effects on Barents Sea ecosystem dynamics. ICES Journal of Marine Science. 2012;69: 1303–1316. doi:10.1093/icesjms/fss063

74. Kędra M, Moritz C, Choy ES, David C, Degen R, Duerksen S, et al. Status and trends in the structure of Arctic benthic food webs. Polar Res. 2015;34: 23775. doi:10.3402/polar.v34.23775

75. Haug T, Bogstad B, Chierici M, Gjøsæter H, Hallfredsson EH, Høines ÅS, et al. Future harvest of living resources in the Arctic Ocean north of the Nordic and Barents Seas: a review of possibilities and constraints. Fish Res. 2017;188: 38–57. doi:10.1016/j.fishres.2016.12.002

76. Pecl GT, Araújo MB, Bell JD, Blanchard J, Bonebrake TC, Chen IC, et al. Biodiversity redistribution under climate change: impacts on ecosystems and human well-being. Science (1979). 2017;355. doi:10.1126/science.aai9214

77. Fredston-Hermann A, Selden R, Pinsky M, Gaines SD, Halpern BS. Cold range edges of marine fishes track climate change better than warm edges. Glob Chang Biol. 2020;26: 2908–2922. doi:https://doi.org/10.1111/gcb.15035

Figure Click here to access/download;Figure;Figures.docx

Table 1: Detailed summary of the effect of best-fit and most reduced GAM predictors on the abundance (Negative binomial distribution) for *C. monstrosa*, *E. spinax* and *G. melastomus* in the Norwegian Sea (variables tested: Depth, Latitude and Distance to the coast in function of the year as well as the Year; interactions between variables are indicated by " : "). Models selected by the Adjusted R^2 (Adj-R^2) and the adjusted proportion of deviance explained (Dv exp). "Estimate" refers to the expected change in the response variable for a one-unit change in the predictor, with the associated "t-value" indicating the strength and direction of the relationship and providing evidence against the null hypothesis of a zero-parameter value. Significance value of statistical tests given as ***: p <0.001.

Model Selection

**Significant predictors**	**AIC**	**Adj R^2**	**Dv exp**
Chimaera monstrosa			
NULL	21835.1		
s(Year, Lat, k = 15)	21478.8		
s(Year, Lat, k = 15) + s(Year, Distance, k = 13)	21304.5	0.460	25.0%
Etmopterus spinax			
NULL	11287.7		
s(Year, Lat, k = 15)	11025.8	0.317	24.4%
Galeus melastomus			
NULL	8846.1		
s(Year, Lat, k = 15)	7800.3	0.546	68.3%

Parametric coefficients

**Variables tested**	**Estimate**	**Std error**	**t value**	***p***
Chimaera monstrosa				
Latitude:Year	4.561	0.066	68.81	***
Depth:Year	5.064	0.070	72.26	***
Distance:Year	5.288	0.072	73.73	***
Year	6.793e-02	9.17e-03	7.41	***
Etmopterus spinax				
Latitude:Year	3.176	0.109	29.15	***
Depth:Year	4.338	0.117	37.05	***
Distance:Year	4.705	0.121	39.06	***
Year	0.054	0.016	3.47	***
Galeus melastomus				
Latitude:Year	4.013	0.453	8.86	***
Depth:Year	3.856	0.119	29.88	***
Distance:Year	3.141	0.115	27.31	***
Year	0.012	0.015	0.78	

Figure 1: Norwegian waters showing the depth contour, the area trawled during the Kysttokt survey since 1995 (yellow) and the relevant ICES ecoregions (demarcated by black lines).

Figure 2: Radar plot with the environmental variables used to define species habitat occupancy (lighter colour) and habitat preference (70% of individuals; darker colour) over the last 26 years. Drawings: *C. monstrosa*, *E. spinax* and *G. melastomus*, CC BY-SA 4.0 Jan Fekjan

Figure 3: Long-term shifts and preferred habitat of *C. monstrosa*, *E. spinax* and *G. melastomus* in the survey area covered between 1995 and 2020. These changes are shown with the (a) Distribution map of *C. monstrosa*, *E. spinax* and *G. melastomus* for 1995 (orange) and 2020 (purple) showing the habitat occupancy (polygons) and abundance (indiv/km^2; dots) as well as the latitudinal centre of gravity in 1995 and 2020 and (b) latitudinal centre of gravity fitted with linear regression. Change through time is written as: slope ± SE. Significant value of statistical tests given as *: $p < 0.05$. Drawings: *C. monstrosa*, *E. spinax* and *G. melastomus*, CC BY-SA 4.0 Jan Fekjan

Figure 4: Long-term abundance shifts in the survey area covered between 1995 and 2020 of the supposed *E. spinax* and *G. melastomus* prey: *A. silus*, *G. argentus*, *M. poutassou* and *T. esmarkii*. These changes are shown with the change of the (a) depth and (b) latitudinal centre of gravity, fitted with linear regression. Only the slopes with a significative p-value are shown. Significant value of statistical tests given as **: $p < 0.01$, *: $p < 0.05$ Drawings: *A. silus*, *G. argentus*, *M. Poutassou* and *T. esmarkii*, CC BY-SA 4.0 Jan Fekjan.

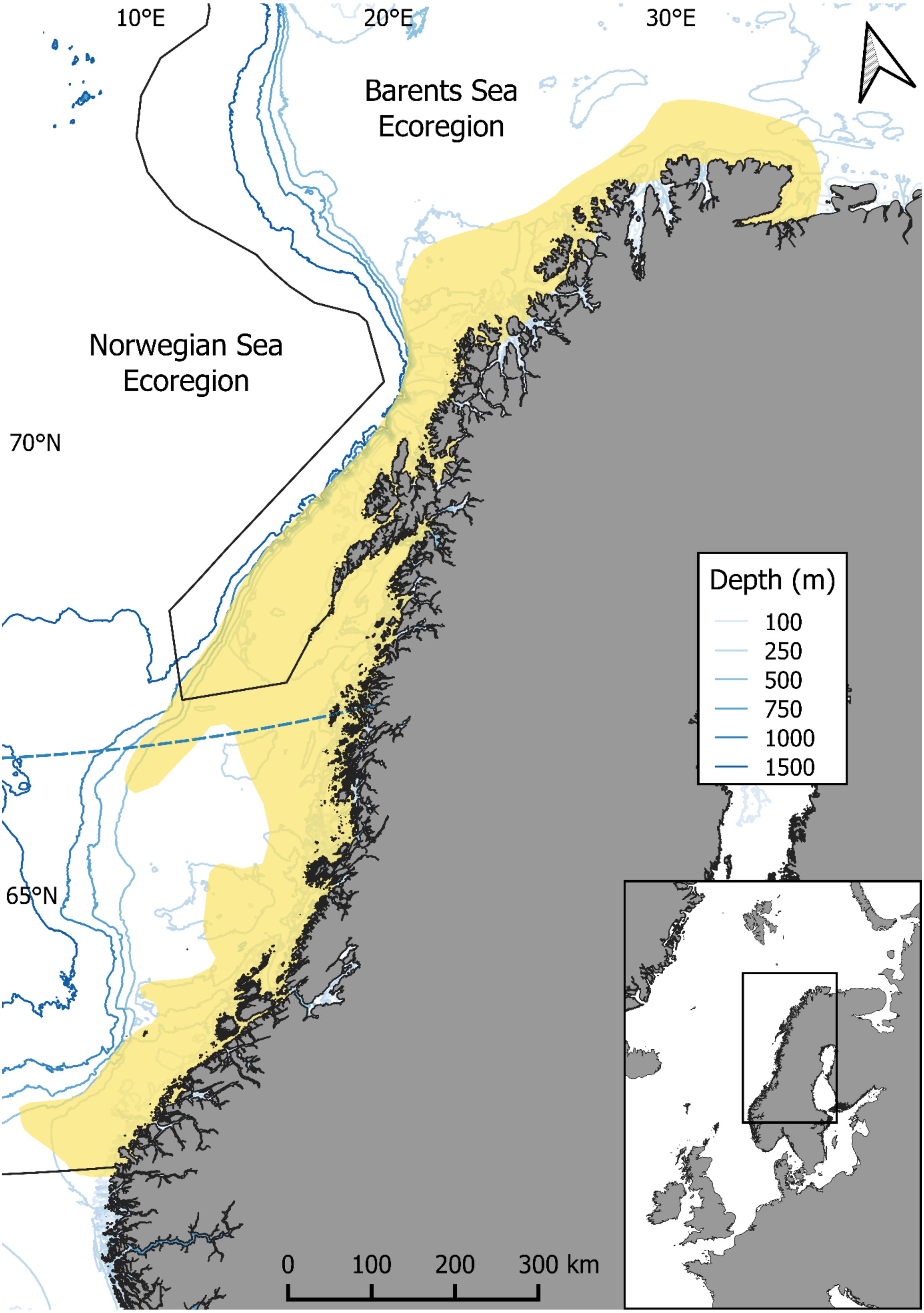
10°E
20°E
30°E
Barents Sea Ecoregion
Norwegian Sea Ecoregion
70°N
65°N
Depth (m)
100
250
500
750
1000
1500
0
100
200
300 km

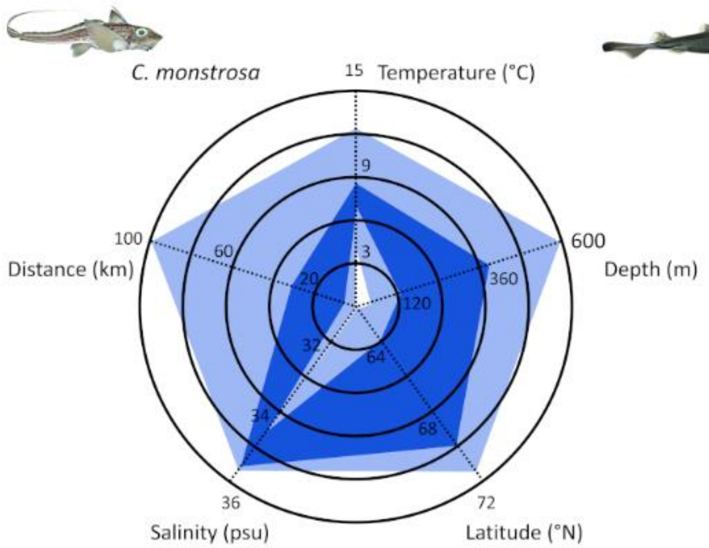
C. monstrosa
15
Temperature (°C)
9
3
100
Distance (km)
60
20
600
Depth (m)
360
120
32
64
34
68
36
Salinity (psu)
72
Latitude (°N)
E. spinax
G. melastomus

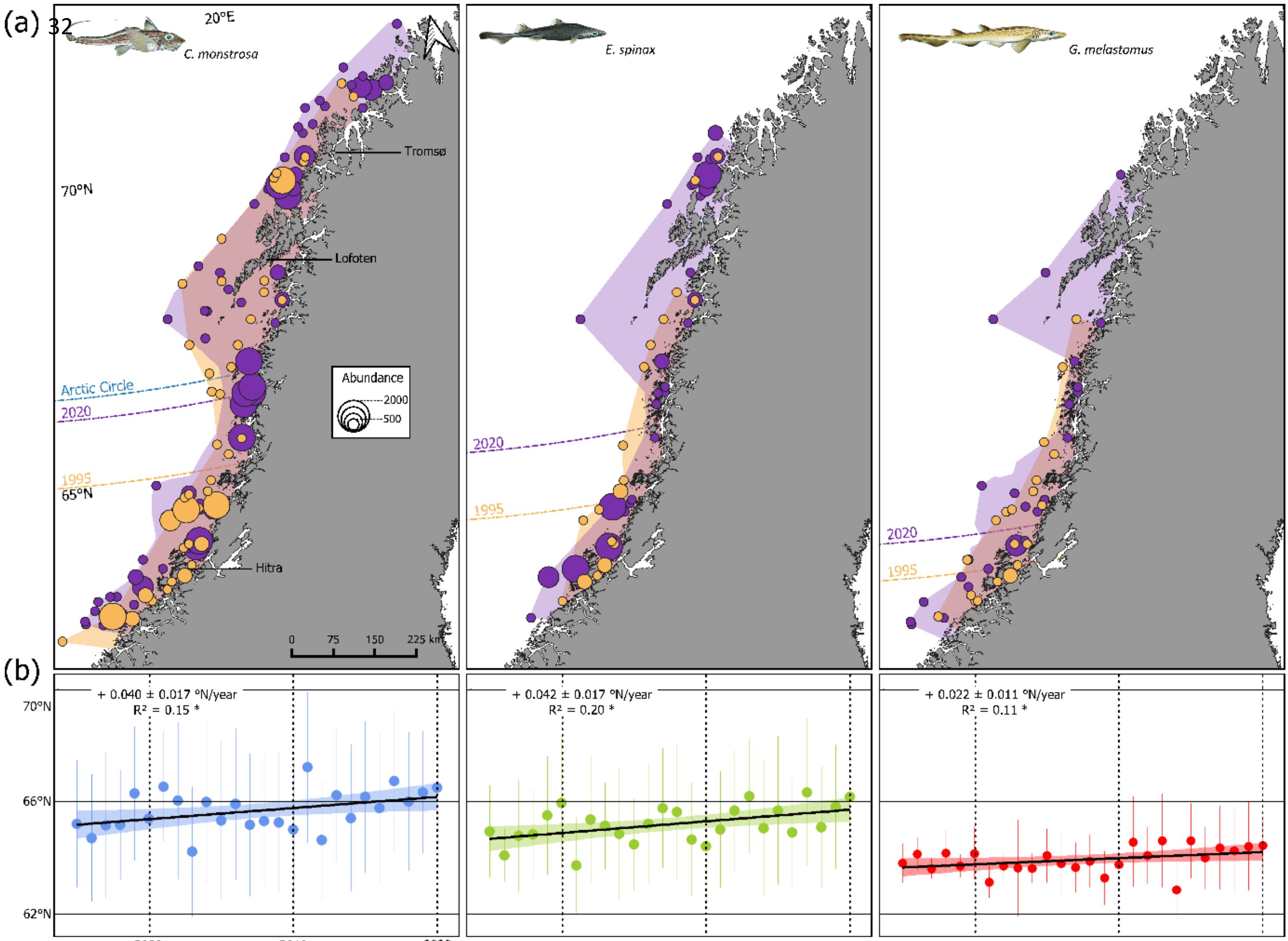
(a)
32
20°E
C. monstrosa
E. spinax
G. melastomus
Tromsø
70°N
Lofoten
Abundance
2000
500
Arctic Circle
2020
1995
65°N
Hitra
0
75
150
225 km
(b)
+ 0.040 ± 0.017 °N/year
R² = 0.15 *
+ 0.042 ± 0.017 °N/year
R² = 0.20 *
+ 0.022 ± 0.011 °N/year
R² = 0.11 *
70°N
66°N
62°N
2000
2010
2020

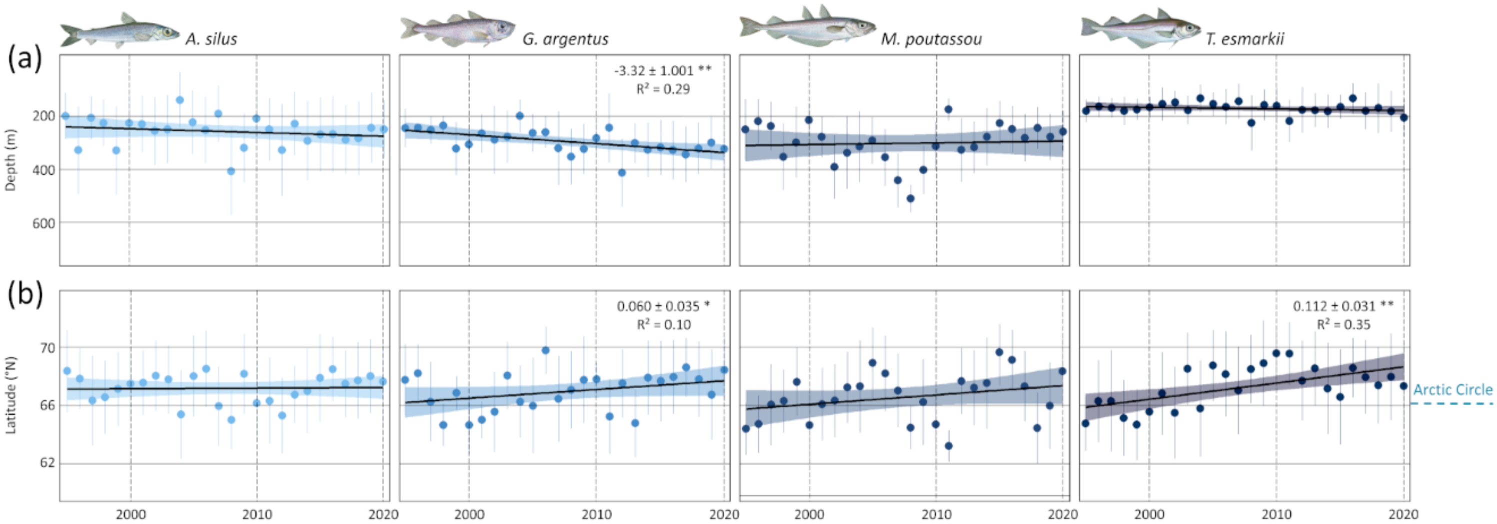
A. silus
G. argentus
M. poutassou
T. esmarkii
(a)
(b)
Depth (m)
200
400
600
-3.32 ± 1.001 **
R² = 0.29
Latitude (°N)
70
66
62
0.060 ± 0.035 *
R² = 0.10
0.112 ± 0.031 **
R² = 0.35
Arctic Circle
2000
2010
2020

Click here to access/download
Supporting Information
Supporting Information.docx
