# Supplementary material for: Moving north: Warmer waters expand populations of deep-water cartilaginous fishes into Arctic waters
Source: PLoS One. 2026 Mar 5;21(3):e0343778. doi: 10.1371/journal.pone.0343778 (PMC12962514; doi:10.1371/journal.pone.0343778)
Supplement: Attachment — Submitted filename: Answer to reviewers.docx [file pone.0343778.s015.docx]

**Answer to reviewers**

We would like to sincerely thank the reviewers, reviewer 1 and Dr. Umberto Scacco for their time, careful evaluation, and constructive feedback, which have greatly contributed to improving the quality and clarity of our manuscript. We have carefully considered all comments and made the corresponding revisions throughout the text. In our responses below, the line numbers refer to the revised clean version of the manuscript (without tracked changes).

**Reviewer 1**

*Major comments*

The study is of high scientific interest. It is well organized and the results are clearly presented. With regard to the methods, the experimental sampling design should be explained, indicating which statistical approach was used to allocate the stations within the investigated depth range.

The main limitation of this study is that it demonstrates the northward shift of the most superficial fraction of the population of the three species considered. In fact, although the study deals with three deep-sea species, the data sampling partially covers the depth range inhabited by these species. Therefore, some measures of distribution and abundance, such as COG, habitat occupancy and preference, relate to the sampled population in the investigated depth range. It is very likely that they don’t represent the population distributed over the whole depth gradient.

I agree that the authors included a section on the limitations of the study. The aforementioned issues regarding the bathymetric range explored also raise the question of whether populations have moved even deeper than estimated.

**Answer:** We thank the reviewer for this constructive comment and for highlighting these important considerations. We have now clarified the experimental sampling design in the Methods section, specifying how stations were distributed across the investigated depth range and which statistical approach was used (see line 97).

We also expanded the discussion of the study’s limitations to address the partial coverage of the depth range occupied by these deep-sea species and the potential underestimation of deeper shifts (see lines 281–283). However, we also wanted to add that in Norwegian waters, these species have been primarily observed within the temperature and depth ranges sampled in the present study (Jac et al., 2022), supporting the relevance of the investigated strata for assessing their distributional changes.

*Specific comments*

**Line 53**: “…ability of the species to exploit new resources…” in presence of predators and competitors in areas of new location.

**Answer**: We agree that the ability of a species to exploit new resources is strongly influenced by biotic interactions in the new environment, including competition and predation. We have therefore revised the sentence to acknowledge these ecological factors (lines 51-54) “The magnitude of these impacts depends on the sensitivity (e.g., thermal tolerance), dispersal capacity (e.g., migratory versus non-migratory), and ability of the species to exploit new resources (generalist versus specialist feeder) and habitats while interacting with resident predators and competitors in newly colonized area.”

**Line 92-94**: Were the data from the two different periods collected using the same sampling design, with the same tool, and within the same depth range?

**Answer**: Yes, data from both periods were collected using the same sampling design, gear type, and within the same depth range. We have clarified this information in the manuscript (lines 95-98).

**Line 96**: 800 ma?

**Answer**: “ma” has been removed

**Line 103**: A bias in the catches is also expected because the investigated depth range does not adequately cover the depth range in which the species are distributed, which is well greater than 1000 m, and 2000 m for E. spinax. Indeed, the species occurrence percentage are low, mostly for G. melastomus and E. spinax. Furthermore, sampling does not allow for the observation of any seasonal variations in the distribution of species throughout the year.

**Answer**: We fully agree that the investigated depth range does not encompass the entire bathymetric distribution of the studied species. We have discussed this important limitation in the Materials and Methods (Line 97) and Discussion (Lines 281-283) sections, highlighting that some bias in the catches is expected because the sampling depth range (down to 500 m) does not fully cover the depths typically inhabited by these species. However, Jac et al. (2022) showed that, in Norwegian waters, these three species are predominantly found within this depth range.

**Line 104-107**: The bathymetric range investigated should be also reported in the text. How were the hauls allocated within the investigated depth range? Which sampling design was adopted?

**Answer**: We added this information in line 97.

**Lines 107-108**: How was the trawl distance measured?

**Answer**: We have rewritten the sentence in line 110-111 to clarify that all information regarding the calculation of trawl distance and abundance is already described in Jac et al., 2022. “Abundance (individuals per km²) was standardised using trawl distance and a mean trawl opening width of 25 m, for more detail see [28].”

**Line 122-127**: However, in figures S3 and S4, temperature and salinity have been reported as depth as 150 m in Bud at 63°N and Eggum at 68°N, and 250 m in Ingøy at 71°N.

**Answer**: This point has been clarified in the revised manuscript. We now specify in lines 127-129 that, although observational data are available down to 300 m at all three sites, the corresponding GLORYS model data extend only to 150 m at Bud and Eggum and to 250 m at Ingøy, due to the model’s simplified bathymetry and relatively coarse horizontal resolution. In addition, a sentence has been added in lines 132-134 to note that the hydrographic data used in our analyses at 150, 250, and 350 m were retrieved from model grid points located slightly farther offshore in deeper areas.

**Line 130-135**: It should be specified that habitat occupancy and preference refer to the sampled population between 30 and 590 m in depth. Unfortunately, the species occurrence percentage are low, mostly for G. melastomus and E. spinax (Table S1).

**Answer**: We agree that habitat occupancy and preference metrics are specific to the sampled population within the depth range of 30–590 m and we addressed that in lines 281-283. However, in the Norwegian waters, these three species are predominantly found within this depth range (see Jac et al., 2022).

**Line 141:** Which depth strata were used to calculate COG which refers to the sampled population between 30 and 590 m in depth?

**Answer**: Yes, the COG was calculated for the sampled population within the depth range of 30–590 m. We have clarified this in the revised manuscript (Lines 147-151).

**Line 164-171**: This approach should be validated by stomach content analysis since during a trawl hauls the distance covered is significant, and different species could be captured far apart from each other.

**Answer**: We thank the reviewer for this comment. Another reviewer also raised a similar point, and we have incorporated this feedback into the revised manuscript. A broader discussion on the feeding ecology of the species has been added in lines 293–329, with a specific section addressing this issue in lines 304–313.

**Line 175-178**: In Figure S6, Eggum is 69°N while in the text is 68°N; temperature variations were assessed up to a depth of 350 m; validation at 71°N was up to a depth of 350 m.

**Answer:** We thank the reviewer for this observation. The latitude has been corrected from 68°N to 69°N in the text (Line 126) to match Figure S6. As clarified in the revised version, the model grid was not deep enough at the three validation sites (Bud, Eggum, and Ingøy). For the validation, comparisons between GLORYS and CTD observations were conducted at the same geographic positions to ensure consistency. However, for the statistical analyses, we prioritized using comparable depth levels across sites; therefore, GLORYS data were extracted from grid points located slightly farther offshore in deeper areas. This explains the apparent discrepancy between positions used for model validation and those used in the analyses.

**Line 180-181**: This is somewhat strange: small variations over the years (much less than 1 degree centigrade) have proven significant, while more marked variations between shallow and deep stations (much more than 2 degree centigrade) are not.

**Answer**: The apparent discrepancy arises from differences in the statistical significance of temporal versus vertical temperature variations. The small but consistent interannual variations shown in Figure S6 were statistically significant due to low within-year variability and long-time series, whereas the larger temperature differences between shallow and deep stations were not statistically significant because of higher spatial variability and fewer samples. Additionally, the autumn time series at 69°N shows a slightly higher p-value than the corresponding 71°N and annual series (Figures S6 and S7), which likely reflects local variability rather than an absence of trend.

**Line 181-182**: “…a marked warming trend over time” apart from the decline around 2020.

**Answer**: We have clarified in the manuscript that the warming trend in waters north of 64°N occurred over most of the study period, but a slight decline was observed around 2020. We also note that this recent variation highlights the importance of further investigation in future studies (lines 191-193).

**Line 216**: What class of Mollusca. Cephalopods are also Mollusca.

**Answer**: Changed it to Gastropoda

**Line 223**: M. poutassou (Blue whiting).

**Answer**: We are not sure that we understand the reviewer’s comment here. In line 230 we have included both the latin and the common name.

**Supporting information**: There are two duplicate figures (S6-S7, S8-S9) in Supporting information.

**Answer**: The figures are not duplicates: S6 and S8 represent the full-year dataset, while S7 and S9 correspond specifically to the September–November sampling season.

**Reviewer 2 (Dr. Umberto Scacco**)

*Major comments*

The paper entitled “Moving North: Warmer waters expand populations of deep-water cartilaginous fishes into Arctic waters.” is an interesting work and it analyses the northward geographical shift of three deep-water species across 26 years data in the Norwegian waters. The paper is well written, concise and tight to the data collected with a proper statistical framework for data interpretation. Deep water mass warming appeared as the first driver in determining such a shift, both directly (i.e. species migrate northward to accommodate their preferential thermal niche) and indirectly (by the shifting distribution of their potential prey as well). On this last aspect, I have some concerns: 1) when authors inferred the trophic driver, they based their hypothesis on the co-occurrence of prey censed in catch mass where targeted cartilaginous species were present, i.e they considered all the prey that could be entrapped by a trawl mesh size of 20 mm. To my knowledge, common prey of G. melastomus and E. spinax can be also smaller than this size as being many taxa of micro crustaceans (amphipods, mysids, euphasids, ostracods, etc), polychaetes (eunicidae, etc), cephalopods (sepiolidae, decapods, octopuses, etc), particularly in early life stages of these species. 2) the co-occurrence of larger prey (fish in particular) can be a valid proxy for the larger specimen (subadults and, the most, adults sharks) only, as ontogenetic shifts in diet have been demonstrated for G. melastomus and E. spinax, chiefly in the Mediterranean basin (prey dimension, and corresponding taxa, increases, and changes, according to the increase in fish dimension, within a generalistic-opportunistic feeding strategy that is a common trait of the species considered (the sharks in particular). Of course, the Mediterranean is a very different environment from Atlantic Ocean with a deep-water bottom temperature being much higher than oceanic corresponding depth strata (Med waters below 200 m depth display a temperature that is maintained constant at 13.5-14.5 °Celsius due to heat pump effect played by the interchange of water masses with the Atlantic; and this temperature threshold is increasing as well in the basin starting form 2000). However, ontogenetic shifts might be considered for these species in the Atlantic Ocean also such that this aspect should be carefully considered. For instance, did the authors collected information on size distribution of the samples, and/or did they perform parallel stomach content analysis on the samples investigated? For instance, comparing size-frequency distributions of different samples collected across the 26 years data set could add important info on changes in population dynamics (if you find that similar size distributions of animals are progressively found northward during the considered time span, then all life stages of the species have been moving northward and so on). Again, if you have performed parallel stomach content analysis, you can have a more complete picture of feeding habits and dietary ontogenetic shifts, i.e. a clearer idea of the prey species (not only bony fish) to consider in the catch mass if the co-occurrence is the only way available to infer the trophic factor as a driver of the northward shift of the species considered. Discussing such aspects could be an added value when claiming the trophic effect as a driver determining the geographical shift.

**Answer:** We thank Dr. Umberto Scacco for these insightful and constructive comments. We agree that this is an important point, particularly regarding the role of trophic dynamics and potential ontogenetic dietary shifts. As no comprehensive study has yet examined the feeding behaviour of *Galeus melastomus*, *Etmopterus spinax*, and *Chimaera monstrosa* in the Norwegian Sea, our approach aimed to provide a first step toward exploring potential trophic influences by examining the co-occurrence of prey species in trawl catches.

We acknowledge, however, that this method has limitations, particularly in representing smaller prey taxa (e.g., micro-crustaceans and polychaetes) and in accounting for ontogenetic shifts in diet. We have now incorporated additional discussion to address these aspects. Specifically, we discuss the size distribution of the sampled individuals and its implications for interpreting trophic drivers in line 263-269, and we expand on the potential dietary shifts during ontogeny and their influence on species’ spatial dynamics in line 294-303.

We also note in the revised manuscript that further studies, including targeted stomach content and stable isotope analyses, are needed to better understand the feeding ecology of these species in the Norwegian Sea and to strengthen inferences about trophic drivers of their northward expansion.

*Specific comments*

**Line 72**: Please consider these species are present in the Mediterranean basin also, within a much warmer environment (13.5-14.5 °C for all deep waters below 200 m depth due to heat-pump effect generated by water circulation and exchang with the Atlantic Ocean). Thermal niche of the species considered appears as wide taking into consideration the difference between the Atlantic and Mediterranean

**Answer:** We thank the reviewer for this insightful comment. However, we found it challenging to make a direct comparison between individuals from the Mediterranean basin and those observed in Norway, given the substantial differences in oceanographic conditions, population dynamics, and demographic structures between these regions.

Nevertheless, we revisited our manuscript and expanded our discussion with this idea in mind, in order to better highlight the potential breadth of the thermal niche of the species considered.

**Line 80:** Pleace Define the acronyms at first appearance

**Answer:** All acronyms have been defined at their first appearance in the manuscript.

**Line 108:** Did the authors collected information on size distribution of the samples along the time series considered? This can have great impact on results. In my opinion, considering abundance expressed as absolute density could hide the effect of fish size on the shifting pattern towards the north

**Answer:** We completely agree with this comment. Length data were also collected during the survey cruises. However, for the purpose of this paper and to maintain a clear narrative, we chose to first focus on the overall population-level patterns before addressing more specific aspects such as length distribution in future analyses.

**Line 132:** spatial variables are different depending on the life stage of each of the species considered, i.e. newborns, juveniles, subadult and adults inhabit different environments

**Answer:** We agree that spatial habitat use can vary across life stages. We have clarified this in the manuscript to acknowledge that different life stages of the studied species may occupy distinct environments, which could influence habitat occupancy and preference metrics (lines 263-269).

**Line 148:** I suggest the authors to consider the mean size of species per sampling operation as a covariate in the analysis. In this way, they will able to evaluate the effect of size in the shifting pattern

**Answer:** We completely agree with this comment. Length data were also collected during the survey cruises. However, for the purpose of this paper and to maintain a clear narrative, we chose to first focus on the overall population-level patterns before addressing more specific aspects such as length distribution in future analyses.

**Line 157**: Please remember that it is an average information due to the fact that each life stage has its own ecological needs thus requiring different environments.

**Answer:** We have discussed that further in the discussion part (line 263-269)

**Line 163**: This point raises some concerns in my opinion. Co-occurrence of prey can inform on potential only interactions between the prey and the three species of sharks and chimaeras analysed for at least two reasons:1) several prey of these species are at microscopic scale (i.e much smaller than mesh size used in the survey); 2) Dietary habit changes during ontogeny, i.e prey are different according to predator size. In the form authors presents their results, co-occurrence can provide an average information at last, as being partial (only co-occurrence of prey larger than 2 cm is considered in the analysis)

**Answer:** We agree with your point and added a discussion about that in line 293-313.

**Line 177**: It is not a validation, it is a verification rather

**Answer:** We now used “to verify” instead of “validates”

**Line 217**: Which are the co-occurred species?? Table 3 is missing (as well as table 2..)

**Answer:** Thank you for noting this. The correct reference is Table **S3**, not Table 3.

**Line 222**: They are some of the prey of larger sharks, not all the prey neither the prey for all the life stages of the cartilaginous species considered

**Answer:** This aspect has now been discussed in line 293-303.

**Line 231**: This result is questionable in my opinion

**Answer:** We were unsure about the specific issue being referred to and therefore may not have fully addressed the concern. We have nevertheless added further details and clarification in line 239-241, and we would be happy to provide additional revisions if the reviewer can elaborate on the comment.

**Line 274**: Please re-think the whole paragraph in the light of previous comments I provided

**Answer:** We have revised the entire paragraph using all your comment about diet; please see the updated version between lines 293-329.

**Line 283**: ...as well as information on the availability of all potential prey will be available

**Answer:** We rewrote entirely the paragraph

**Line 286-287**: ...and many other taxa, such as amphipods, mysids, ostracods, etc.). Cephalopds also are constant component of diet in these two species, i.e. Heterotheutys dispar (a sepiolidae) is a common prey for G. melastomus, as well as the larger Histiotheutys spp is a common prey for E. spinax

**Answer:** We rewrote entirely the paragraph

**Line 294**: If authors are unable to put into the statistical analysis my suggestions about size distribution of the samples across years along the entire time series and about dietary shifts during ontogeny, they have also the possibility to argument such limitations in this paragrapph

**Answer:** We have incorporated additional paragraphs to address these points. Specifically, we now discuss the size distribution of samples in line 263-269 and the potential dietary shifts during ontogeny in line 294-303
