# Supplementary material for: Moving north: Warmer waters expand populations of deep-water cartilaginous fishes into Arctic waters
Source: PLoS One. 2026 Mar 5;21(3):e0343778. doi: 10.1371/journal.pone.0343778 (PMC12962514; doi:10.1371/journal.pone.0343778)
Supplement: Attachment — Submitted filename: Answer_to_reviewers_auresp_2.docx [file pone.0343778.s016.docx]

**Answer to reviewers**

We sincerely thank both reviewers, Reviewer 1 and Dr. Umberto Scacco, for their time, careful evaluation, and constructive feedback, which have greatly contributed to improving the clarity and quality of our manuscript. We have carefully addressed all comments and made corresponding revisions throughout the text. Line numbers in our responses refer to the revised clean version of the manuscript (without tracked changes).

**Reviewer 1**

*Comments*

The work has been substantially improved, although some limitations, adequately addressed by the authors, remain.

In particular, the sampling design adopted is still unclear. It was not reported in line 97. What approach (random-stratified, systematic, other) was used to allocate the stations along the whole bathymetric gradient investigated? This is important for calculating the COG. In fact, if the stations have been allocated unbalanced along the whole depth gradient, the COG value may be unreliable.

**Answer:** We thank the reviewer for highlighting the need to clarify our sampling design. In the revised manuscript, we have specified that the stations were allocated using a systematic stratified design along the entire bathymetric gradient, covering shallow, intermediate, and deep strata (line 101-103). We have also clarified in line 174-176 that this approach ensures a balanced representation of depths across the study area, minimising potential bias in the calculation of the centre of gravity (COG).

**Reviewer 2 (Dr. Umberto Scacco**)

*Comments*

I think the revised version of the paper has addressed almost all my concerns arose in the first review round. However, I also noticed that the introduction should need a brief paragraph contextualizing and stressing the importance of trophic drivers, which is later considered one of the two main factors determining northward shift of species investigated. I think this paragraph should contain general information on what is already known on species trophic habit of the investigated species at the regional but also at larger scale than regional where these species are present as well. Maybe, this could be done after line 54 of the revised introduction, but authors can place such a part wherever convenient throughout the text.

**Answer:** We thank Dr. Umberto Scacco for this suggestion. In response, we have added a paragraph in the Introduction (line75-81) to contextualize the role of trophic interactions in shaping species’ distributions and northward shifts. Specifically, we now describe the diets of *Chimaera monstrosa*, *Etmopterus spinax*, and *Galeus melastomus* at both regional and broader Northeast Atlantic scales, highlighting that prey availability may facilitate or constrain northward movements. This addition emphasizes trophic drivers as a key factor alongside environmental variables in determining species’ range shifts. It can be read as follow “Trophic interactions further shape their distribution and range shifts: *C.* *monstrosa* feeds primarily on benthic crustaceans, molluscs, and small demersal fishes [29,30], *E. spinax* consumes mesopelagic fishes, cephalopods, and crustaceans [31–33], and G*. melastomus* feeds opportunistically on small fishes, cephalopods, and crustaceans [32,33]. These consistent dietary patterns across the Northeast Atlantic suggest that prey availability at regional and broader scales may facilitate or constrain northward movements.”
